# Intrinsically disordered proteins play diverse roles in cell signaling

**DOI:** 10.1186/s12964-022-00821-7

**Published:** 2022-02-17

**Authors:** Sarah E. Bondos, A. Keith Dunker, Vladimir N. Uversky

**Affiliations:** 1 Department of Molecular and Cellular Medicine, Texas A&M Health Science Center, College Station, TX 77843 USA; 2 Center for Computational Biology and Bioinformatics, Department of Biochemistry and Molecular Biology, Indiana University School of Medicine, Indianapolis, IN 46202 USA; 3 Department of Molecular Medicine and USF Health Byrd Alzheimer’s Research Institute, Morsani College of Medicine, University of South Florida, Tampa, FL 33612 USA; 4 Institute for Biological Instrumentation of the Russian Academy of Sciences, Federal Research Center “Pushchino Scientific Center for Biological Research of the Russian Academy of Sciences”, Pushchino, Moscow Region, Russia 142290

**Keywords:** Cell signal amplification, Integration, Differentiation, Propagation, Specificity

## Abstract

**Abstract:**

Signaling pathways allow cells to detect and respond to a wide variety of chemical (e.g. Ca^2+^ or chemokine proteins) and physical stimuli (e.g., sheer stress, light). Together, these pathways form an extensive communication network that regulates basic cell activities and coordinates the function of multiple cells or tissues. The process of cell signaling imposes many demands on the proteins that comprise these pathways, including the abilities to form active and inactive states, and to engage in multiple protein interactions. Furthermore, successful signaling often requires amplifying the signal, regulating or tuning the response to the signal, combining information sourced from multiple pathways, all while ensuring fidelity of the process. This sensitivity, adaptability, and tunability are possible, in part, due to the inclusion of intrinsically disordered regions in many proteins involved in cell signaling. The goal of this collection is to highlight the many roles of intrinsic disorder in cell signaling. Following an overview of resources that can be used to study intrinsically disordered proteins, this review highlights the critical role of intrinsically disordered proteins for signaling in widely diverse organisms (animals, plants, bacteria, fungi), in every category of cell signaling pathway (autocrine, juxtacrine, intracrine, paracrine, and endocrine) and at each stage (ligand, receptor, transducer, effector, terminator) in the cell signaling process. Thus, a cell signaling pathway cannot be fully described without understanding how intrinsically disordered protein regions contribute to its function. The ubiquitous presence of intrinsic disorder in different stages of diverse cell signaling pathways suggest that more mechanisms by which disorder modulates intra- and inter-cell signals remain to be discovered.

**Graphical abstract:**

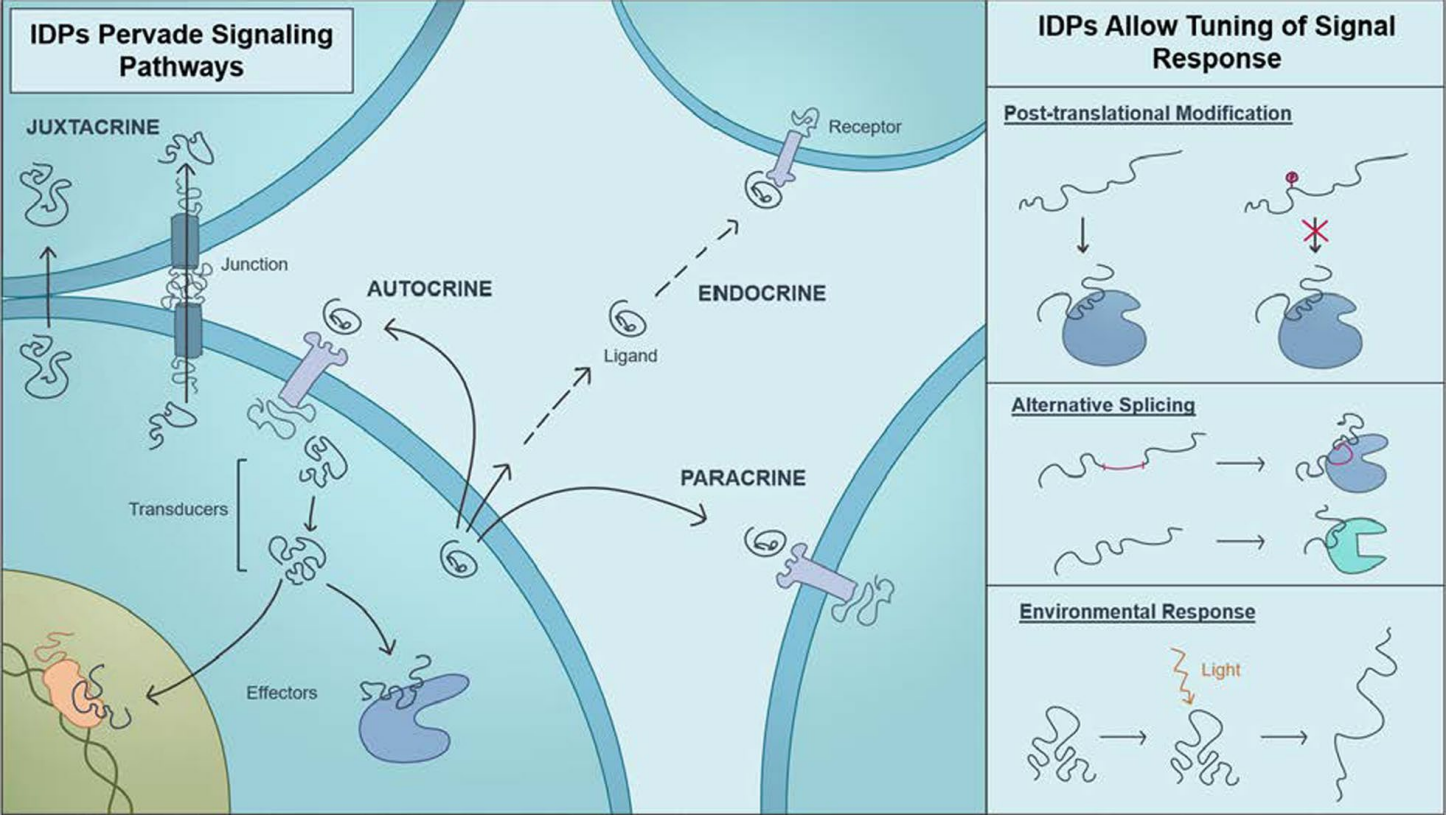

## Background

A wide variety of signaling pathways form a vast communication network that regulates basic cell activities and coordinates biological functions within an organism. Some signaling pathways even allow chemical communication between different organisms or species. Cells communicate with each other to coordinate a variety of functions between cells, tissues, and organs, and all cells must respond to environmental cues. Many mechanisms have evolved to transmit these signals. Extra-cellular signaling involves releasing or displaying any of a variety of chemicals, which are specifically recognized by a receiving cell that then activates an intracellular signal transduction pathway to respond to the signaling event. These events typically rely upon a series of protein–ligand and protein–protein interactions. Such signals must effectively propagate across long distances and even across barriers, such as the cell membrane. Each step in the signaling pathway must be highly specific, both to preserve the integrity of the signaling pathway’s ability to generate a reliable response, and to ensure that the pathway is not erroneously activated. Because a cell’s response to a signal must be transitory, each of these molecular interactions must be reversible. Ligand recognition must both augment and stabilize the response to the signal. These requirements of cell signaling impose unique, and often conflicting requirements on the proteins that constitute the signaling pathways, such as signal integrity versus crosstalk. These apparent conflicts extend to the individual interactions that propagate the signal, an apparent conflict between specificity (large interaction interface) versus reversibility (low free energy of interaction).

**Fig. 1:**
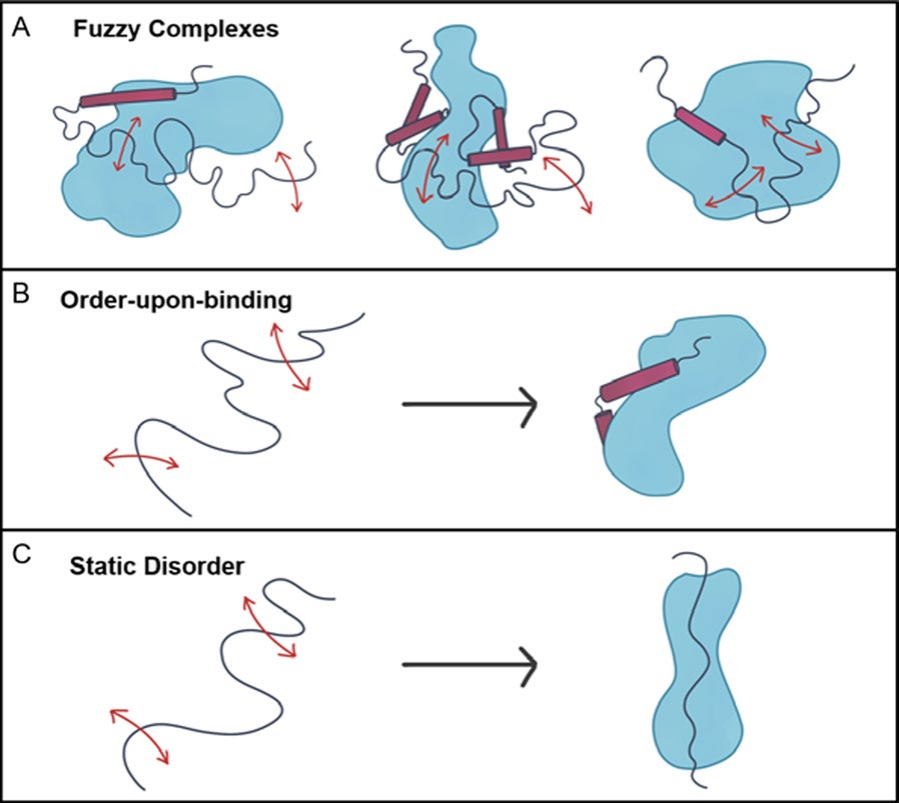
When binding a partner protein, intrinsically disordered regions can adopt multiple unstructured or structurally ambiguous topologies to form fuzzy complexes (**A**), fold to create stable secondary/tertiary structure (**B**) or adopt an unstructured yet static conformation (**C**)

These conflicting needs have been resolved to a large extent by including intrinsically disordered proteins in cell signaling pathways through biological evolution. Intrinsically disordered proteins (IDPs) or intrinsically disordered regions (IDRs) of proteins fail to fold into stable, defined structures as free monomers. Numerous functional advantages of intrinsic disorder are outlined in dedicated studies (e.g., see [1–7]. Many IDPs/IDRs are capable of at least partial ordering upon interaction with specific partners [7–18]. Upon binding of an IDP/IDR to a signaling partner, the free energy required to bring about the disorder-to-order transition subtracts from the interfacial, contact-free energy, resulting in a highly specific interaction that can be combined with a low net free energy of association [3, 18]. Thus binding-induced folding decouples binding affinity from specificity, enabling cell signaling to be reversible. Some IDPs/IDRs may remain unfolded and dynamic even in the bound state [19–23], creating fuzzy complexes (Fig. 1) [24, 25]. An extreme example of this behavior is provided by two highly disordered human proteins, histone H1 and its nuclear chaperone prothymosin-α, which form a picomolar affinity complex, but in which they preserve completely their structural disorder, their long-range flexibility, and their highly dynamic character [19]. Whether or not stable structure results from the interaction, ligand or protein interactions shift the conformational ensemble of the IDP/IDR, linking protein function to binding. Because some signaling IDPs/IDRs lack stable (secondary) structure, the energetic barriers between the bound and free states are low, allowing disordered regions to act as reversible, extremely sensitive sensors. In addition to chemical signals, environmental conditions may also instigate signaling pathways that detect mechanical stress, light, pH, or redox potential [26]. Once the signal is received, the response can be propagated over hundreds of nanometers from the cell membrane to the nucleus, a feat that could dilute the signal. The low energetic barriers that characterize the transition between active and inactive states in intrinsically disordered proteins help shift the equilibrium toward the active state [27, 28]. When protein interaction sites are located within intrinsically disordered regions, the protein associations required to propagate cell signaling pathways are significantly accelerated [29]. Furthermore, the protein–protein interactions that propagate the intracellular signal often allosterically trigger post-translational modifications (PTMs) [30]. The combination of allosteric regulation with a catalytic output (e.g. kinase activity) can also amplify the response to the signal, ensuring it successfully reaches the nucleus [31]. Indeed, the presence of intrinsically disordered regions increases the potential for allosteric regulation [32, 33]. Finally, disordered proteins provide many avenues for integrating multiple signaling pathways [27], including providing a scaffold that binds proteins from multiple pathways [34], regulating multiple disordered substrates through PTMs, and varying pathway components through alternative mRNA splicing [35–37]. This combination of regulatory and environmental factors sometimes modulates protein behavior in a rheostat-like manner [30].

The combined use of intrinsic disorder, alternative splicing and PTM widely enables the context-dependent orchestration of signaling in developmental biology and other complex processes [38, 39]. The mRNA involved in alternative splicing shows a strong preference to code for disorder rather than for structure (with structured-domain-encoding exon skipping being the main exception to this preference); adding and deleting protein segments is much less disruptive in IDRs than in structured regions [40]. Likewise, phosphorylation [41] and many but not all other PTMs [42] show a strong preference to be localized in IDRs, likely because flexibility enhances the ability of a motif, destined for PTM addition, to enter and bind to the active site of the corresponding enzyme. The concentration of both PTMs and segments encoded by alternative splicing within IDRs suggest these two sequence modifications may be co-localized, providing an opportunity to integrate two different regulatory inputs. Indeed, the signaling functions carried out by G protein-coupled receptors (GPCRs), which are transmembrane proteins; the nuclear factors of activated T cells (NFATs), which are transcription factors; and the Src family kinases (SFKs), which are signaling enzymes, are all modulated by the collaboration of PTMs and alternative splicing that map to the same IDRs [39]. Computer searches suggest that PTMs and alternative splicing are commonly studied individually for their effects on cell signaling, but they are rarely considered together. The supplemental data in Zhou et al. shows the results of such computer searches for 11 signaling pathways and 28 well characterized signaling proteins of various kinds [39]. The strong preferences of PTMs and protein segments encoded by alternative splicing to be colocalized in IDRs means that alternative splicing and PTMs will commonly work together to orchestrate signaling. From these considerations, this system has been called the IDP-AS-PTM toolkit [39].

Different combinations of PTMs can integrate information from multiple signaling pathways to create diverse outcomes. Indeed, multiple signaling pathways reversibly add different PTMs to the disordered tails of histone proteins [43]. The resulting collection of PTMs constitutes a “histone code” that elicits unique gene regulatory responses [44]. These differences can carry over to following generations, indicating that these multiple PTMs provide the basis for an epigenetic mechanism. Different signaling outcomes based on different combinations of PTMs have been observed for additional proteins [45–51]. These various multiple PTM-based signaling systems have been given different names, including PTM code [45], which will be used herein. Whether any of these additional applications of the PTM code result in epigenetic effects is unknown to these authors. Experiments and predictions indicate that for all of these proteins the multiple PTMs are located in IDRs [52]. Thus, IDRs are an important feature of both the PTM code and the epigenetic effects observed for the histone code. Furthermore, the supplemental data in Zhou et al., 2018 show that all of these proteins undergo alternative splicing, suggesting the possibility that alternative splicing could be a previously unsuspected regulator of the epigenetic effects resulting from the histone code [39]. Finally, histone tails may be accessible or may adhere to the nucleosome and be inaccessible for protein interactions, demonstrating that the context provided by the nucleosome is also a key contributor [53].

Undoubtedly, understanding and manipulating cell signaling pathways requires clearly defining the roles of IDPs and IDRs. The problem is further complicated by the nesting doll (Matryoshka)-like organization of the cellular signaling, which represents a complex network of networks, with even a single node in a protein–protein interaction network representing a multileveled network itself [54]. Here, at the lowest level, different segments of polypeptide chain form secondary structure elements that represent local networks of hydrogen bonds and residue-residue interactions. The next level of the protein intramolecular network is formed by interactions between the secondary structure elements, which are the local networks themselves. Next, proteins domains are higher level networks that are generated via interactions between these second-tier networks. A functional monomeric protein represents seemingly highest-level network that includes inter-domain interactions and interactions between domains and second-tier networks. Formation of an oligomeric protein or engagement in the temporary protein–protein interactions within the signaling network would require a new level of inter-subunit interactions, where the inter-protein interaction network might include interactions between the networks of various lower levels [54]. This manuscript is part of a collection, the goal of which is to highlight the crucial role of intrinsic disorder in cell signaling, introduce scientists to the basic concepts, common experimental approaches, and diverse molecular mechanisms that characterize each field in the hopes of increasing understanding and promoting further interdisciplinary studies. This review will (1) summarize best practices for identifying intrinsically disordered regions, (2) highlight the variety of cell signaling pathways that employ disordered proteins, and (3) identify examples of currently known molecular mechanisms implemented by these proteins and regions.

## Approaches to examining intrinsic disorder

Under physiological conditions, ordered proteins are known to possess unique three-dimensional (3D) structures, in which each atom of the polypeptide chain has a unique position in space. These structures are determined by a vast number of intrachain non-covalent side chain-side chain, side chain-backbone, and backbone-backbone interactions. Such structure-determining conformational interactions include hydrophobic interactions, hydrogen bonding, Van der Waals forces, and ionic/electrostatic interactions. These interactions have different physico-chemical natures and their strengths depend on the amino acids involved and on the peculiarities of the interactions of a polypeptide chain with solvent. Kinetically, the self-organization of a protein molecule from its unfolded state to a unique 3D structure represents a complex sequential process accompanied by the formation of several partially folded intermediates [55–62]. Furthermore, for many ordered proteins, various partially folded intermediates, such as more extended pre-molten globules or compact molten globules [59, 62–69] can be stabilized by changes in the environment even under the equilibrium conditions. On the other hand, intrinsically disordered proteins (IDPs) or intrinsically disordered protein regions (IDRs) do not have stable 3D-structures under physiologic conditions, existing instead as highly dynamic conformational ensembles, members of which interconvert on a number of timescales. It seems that due to the peculiarities of their amino acid sequences, folding of IDPs/IDRs under physiological conditions is halted at different stages, creating extended-disordered conformations (native coils or native pre-molten globules) or collapsed-disordered conformations (native molten globules) [3, 11, 69].

The situation is further complicated by the fact that not only the entire protein, but its different parts can be disordered to different degrees. As a result, IDPs are not homogeneous, but represent a very complex mixture of a broad variety of potentially foldable, partially foldable, differently foldable, or not foldable segments [70–72]. More globally, a typical protein represents a highly heterogeneous entity with a mosaic spatiotemporal structural organization containing foldons (independent foldable units of a protein), inducible foldons (disordered regions that can fold, at least in part, due to the interaction with binding partners), inducible morphing foldons (disordered regions that can differently fold upon interaction with different binding partners), non-foldons (non-foldable protein regions), semi-foldons (regions that are always in a semi-folded form), and unfoldons (ordered regions that have to undergo an order-a to-disorder transition to become functional) [70, 73–75].

Again, this structural heterogeneity is determined by specific features of the amino acid sequences of IDPs/IDRs. In fact, in comparison with ordered proteins and domains, most IDPs/IDRs are systematically depleted in order-promoting residues (Cys, Trp, Tyr, Phe, Ile, Leu, Val, and Asn), while being simultaneously enriched in disorder-promoting residues (Pro, Arg, Gly, Gln, Ser, Glu, Lys, and Ala) [4, 6, 76–82] and commonly containing repeats [83–86]. In other words, the amino acid alphabet of IDPs/IDRs is decreased in comparison with that of ordered proteins and domains and such disordered proteins/regions are characterized by the reduced informational content of their amino acid sequences [71]. These traits make the location of IDPs/IDRs within an amino acid sequence predictable and accurate [79, 87–91]. In a recent experiment, structure/disorder prediction algorithms were applied a set of 646 proteins with regions of structure and disorder unknown beforehand to the researchers who carried out the predictions. The top three predictors exhibited balanced accuracies on this dataset ranging from 76 to 80% [92]. Since various attributes and computational approaches can be utilized for the development of computational tools for predicting intrinsic disorder from protein sequence, it is not surprising that there are multiple computational tools that one can use to (a) evaluate the overall disorder status of a query protein and (b) analyze the peculiarities of distribution of the intrinsic disorder predisposition within its amino acid sequence [93]. Detailed description of these tools is outside the scope of this article. Interested readers can find related information in a number of dedicated reviews (e.g., [94–102]).

Application of these algorithms to various proteomes indicate that IDPs and IDRs are abundantly present in any given proteome, although eukaryotes have much more disorder than prokaryotes [3, 103–107]. In one such study, the proteomes of a collection of archaea and eubacteria are predicted to have about 15–30% of their encoded residues to be intrinsically disordered, while, in a collection of eukaryotic proteomes, 30–50% of the encoded residues are predicted to contain in IDPs plus IDRs [107].

Contrarily to mostly catalytic, transport, and protein interaction domain functions of ordered proteins and domains [78, 108, 109], IDPs/IDRs are typically involved in signaling, regulation, recognition, and control of various cellular pathways [10, 11, 14, 109–111]. In addition, by changing its shape, a single disordered protein or region can readily bind one-at-a-time to multiple divergent, targeted partners to associate with many different targets [4, 112–117]. Furthermore, sites of various catalytically driven PTMs, such as hydroxylation, acetylation, glycosylation, ubiquitination, SUMOylation, methylation, phosphorylation, etc. and sites of proteolytic attack are often associated with regions of intrinsic disorder [3, 52]. One should also remember that in addition to their structure-based catalytic activities, many enzymes contain functionally important IDRs [118].

Often, disorder-to-order-transition-based recognition is mediated by the specific functional elements known as molecular recognition features (MoRFs) [119–122], which are implicated in important biological processes, including regulation and signaling [123]. Importantly, such disorder-based binding sites can be predicted. For example, α-MoRF-Pred and α-MoRF-PredII algorithms identify disordered regions that have a propensity to become α-helical upon partner binding [119–121]. Another computational tool, MoRFPred, can identify all MoRF types (α, β, irregular, and complex) [123]. Yet another tool, ANCHOR, predicts disordered binding regions (DBRs) located in IDPs or IDRs by identifying segments in a generally disordered region that cannot form enough favorable intrachain interactions, but still have the capability to energetically gain by interacting with a globular partner protein [124, 125]. Some other tools for finding disorder-based binding sites include MFSPSSMpred [126], MoRFCHiBi [127–129], Retro-MoRFs [130], MoRFPred-plus [131, 132], OPAL [133], length-specific MoRF predictor OPAL+ [134], ensemble deep convolutional neural network-based MoRF predictor en_DCNNMoRF [134], SPOT-MoRF [136], MoRF_MPM_ [137], and MoRFPred_en [138].

An alternative approach has been to identify DBRs from their sequence patterns or motifs. Examples of this approach include eukaryotic linear motifs (ELMs) [139, 140], linear motifs (LMs) [141], and short linear motif (SLiMs) [142–144]. One advantage of linear motifs compared to MoRFs is that example binding partners are often known for the former but typically not for the latter. DBRs identified by sequence patterns and MoRFs identified by localized features within regions predicted to be disordered are essentially equivalent, differing mainly in how they are identified.

Protein–protein interactions have been further studied by high throughput methods such as the yeast 2 hybrid assay [145]. Such high throughput methods have taken us from function-specific pathways developed using one-by-one research methods to the more comprehensive proteome-wide protein–protein interaction networks. In these networks, most proteins bind to very few or even just one protein partner, while very few proteins, called hubs, bind to many partners [146]. Hub protein deletion is typically much more deleterious than the deletion of non-hubs [147]. These hub proteins were suggested to have special features enabling them to readily form new connections over evolutionary time, and, indeed, enabling them to be able to form alternative connections with different partners [148], thus leading to the question: what special feature gives hub proteins the ability to bind to multiple protein partners and to readily evolve so as to bind to new partners [149]? IDRs were proposed to be this special feature [10, 117].

Hub proteins have been shown to employ IDRs for multiple partner binding using two completely different mechanisms [10]: namely, (1) one DBR in one IDR associates individually over time with many different structured partners (one-to-many binding); and (2) many different DBRs in one or more IDRs associate individually with one structured partner (many-to-one binding). The p53 transcription factor is an exemplar of the former [117], while the 14-3-3 protein interaction domain and the Sarc Homology 2 (SH2) interaction domain are examples of the latter [117]. Collections of interactions pairs of both types have been studied, revealing the general importance of IDR conformational flexibility for enabling one IDR to bind individually to multiple partners (one-to-many binding) [114] or for enabling many different IDRs to bind individually to a single partner (many-to-one binding) [112].

There are also computational tools for predicting disorder-based sites responsible for interaction with RNA and DNA (e.g., DisoRDPbind [150–152], and regions associated with multiple PTM sites [52]. Advantages and disadvantages of many of these tools were systematically analyzed in several recent studies [101–153], and another comprehensive review shed some light on “a new page in protein science, where molten keys operate on melted locks and where conformational flexibility and intrinsic disorder, structural plasticity and extreme malleability, multifunctionality and binding promiscuity represent a new-fangled reality” [154].

Associated with a multitude of computational tools for finding intrinsic disorder in proteins and predicting various aspects of disorder-based functionality is a huge arsenal of experimental approaches that allow focused investigations of the structures and conformational dynamics of IDPs/IDRs (reviewed in [98, 155–159]) and for the analysis of their functions [154]. These tools are too numerous to be even briefly considered here. This is not surprising, since a protein molecule is a complex entity with multi-levelled structural organization, and since multiple experimental approaches are elaborated for the analysis of protein structure (and lack thereof) in general and for specifically examining the different levels of protein structural hierarchy.

## IDPS/IDRS pervade signaling pathways in all kingdoms of life

Cell signaling requires transient yet highly specific protein interactions, signal sensitivity, signal integration and amplification, and mechanisms to activate/inactivate the entire process in response to changes in the chemical or physical environment. Intrinsic disorder provides the functional diversity, interaction specificity, and regulatory mechanisms that cell signaling processes require. Not every protein in every cell signaling cascade includes intrinsic disorder, and disorder is more prevalent in some cell signaling pathways than others [160]. Nevertheless, intrinsically disordered proteins are present in diverse cell signaling cascades in all kingdoms of life. Increased complexity in eukaryotes creates an increased need for cell signaling and regulation [120].

Aside from the well-studied mammalian cell signaling pathways, disorder is also present in signaling pathways in bacteria [161], algae (see CP12 discussion under redox signaling, below) [26], fungi [34], and plants (see UVR8 discussion under light signaling, below) [162–164]. In bacteria, changes in environment are often detected through protein activity sensing, in which sensing is mediated by post-translational modification of intrinsically disordered regions or unfolding of signaling proteins [165]. A variety of proteins can serve as activity sensors, including enzymes and membrane channel proteins. For example, aconitase serves as an enzyme in the Krebs/citric acid cycle [165]. However, in a variety of bacteria species aconitase can also undergo an environmentally-triggered conformational change that switches its activity from energy generation to post-translational regulation of metabolism and motility. When oxidation or iron depletion destroys the iron-sulfur clusters in aconitase, this enzyme partially unfolds and binds to specific mRNA sequences. The nature of the mRNA determines whether aconitase binding increases or decreases mRNA stability, and thus increases or decreases mRNA translation of the respective proteins. The changes in concentration of the target proteins, for example, FlgR and urease, regulate metabolism and bacterial motility.

Although yeast contain many disordered proteins involved in signaling, a particularly interesting example is the hub protein Killer Nine Resistant 4 (Knr4), which links cell wall synthesis and cell wall integrity with morphogenesis and cell cycle progression [34]. Both the cell wall integrity pathway and the calcineurin pathway are needed to regulate cell wall synthesis and maintenance in response to stress. Knr4 binds the Slt2 MAP kinase in the cell wall synthesis pathway and can repress all of the chitin synthase genes. Knr4 also binds calcineurin in the calcium-calcineurin pathway, and loss of the *knr4* gene makes cells hypersensitive to calcium. Knr4-calcineurin participate in multiple cell cycle checkpoints, coupling cell division, and bud growth, and daughter cell size. While Knr4 phosphorylation is required for binding to at least some of its protein partners, including Slt2 MAP kinase, phosphorylation also appears to facilitate Knr4 degradation. The network of protein interactions formed by Knr4 is conserved among fungi.

## IDRS/IDPS pervade pathways that respond to a wide variety of signals

IDPs/IDRs are found in pathways initiated by a variety of molecular signals, ranging in size from single-atom ions, small molecules such as steroid hormones, and biomacromolecules like nucleic acids and proteins [166, 167]. The examples of intrinsically disordered proteins described below highlight many of the mechanisms by which IDPs/IDRs fulfill the needs of cell signaling pathways.

*Ions* Multiple proteins in the calcium signaling pathway are intrinsically disordered. First, calcium channels permit the passive transport of Ca^2+^ into a cell, either by voltage-gated and/or ligand-gated mechanisms. In the spine, the N-methyl-D-aspartate (NMDA) receptor is a tetrameric Ca^2+^ ion channel which induces different cellular responses—long-term potentiation or long-term depression—based on the intracellular concentration of Ca^2+^ and frequency of stimulation by which it is activated [168]. NMDA receptor activation requires membrane depolarization, which prevents Mg^2+^ from blocking NMDA receptor activity [169], and binding by both glutamate and either glycine or serine. Thus, the NMDA receptor is sensitive to both voltage and ligands. Upon entry into a nerve cell, Ca^2+^ binds calmodulin, and either increases synapse response (long-term potentiation) or decreases synapse response (long-term depression). Protein complexes formed by the intrinsically disordered intracellular tail of the NMDA receptor modulates the cellular response to NMDA activity. High concentrations of Ca^2+^ activate calmodulin-dependent kinases, such as calmodulin-dependent kinase II, and thus long-term potentiation, whereas low concentrations of calcium activate the only phosphatase, calcineurin, and thus stimulate long-term depression [169–173].

The intrinsically disordered long C-terminal tail of the NMDA receptor also regulates calcium signaling by altering the properties of the channel and Ca^2+^ trafficking through the channel [169]. This tail also serves as a scaffold to assemble the downstream signaling proteins, including calmodulin, kinases, and calcineurin. The close proximity of these factors boosts signaling throughput, and the length of the tail defines the search radius for interacting proteins. This reach is varied by alternative splicing [170] and calpain digestion [169], while PTMs regulate nearly every aspect of protein function, including stability, trafficking, recycling, protein interactions, and calpain digestion [169, 171]. The presence of intrinsic disorder in proteins enables regulation by a combination of protein interactions, alternative splicing, and PTMs, which in turn allows multiple signals to fine-tune cell protein function and regulate signal strength, which in turn determines the cellular response [172]. This protein appears to be a candidate for using the IDP-AS-PTM Toolkit for regulating its signaling complexity as described earlier for three other proteins [39].

Calcineurin provides a second example of the role of intrinsic disorder in ion signaling, which is described detail by Trevor Creamer in this collection [173]. Together, calmodulin and calcineurin act as an intracellular Ca^2+^ sensors and responders [173–180]. Both proteins bind calcium, and then each other to form an active phosphatase. Calcineurin activity is also linked to its structure which can occupy three unique states [173]. In the inactive state, a regulatory domain within calcineurin is protected from proteases, and thus presumably folded. In the presence of Ca^2+^ but the absence of calmodulin, the regulatory domain is unfolded and solvent-exposed. Calmodulin binding to the regulatory region induces folding to a helix, and release of an auto-inhibitory domain from the active site of calcineurin. Such coupling of protein (or ligand) binding with protein folding is another common feature of intrinsically disordered proteins [12, 174, 175].

**Fig. 2:**
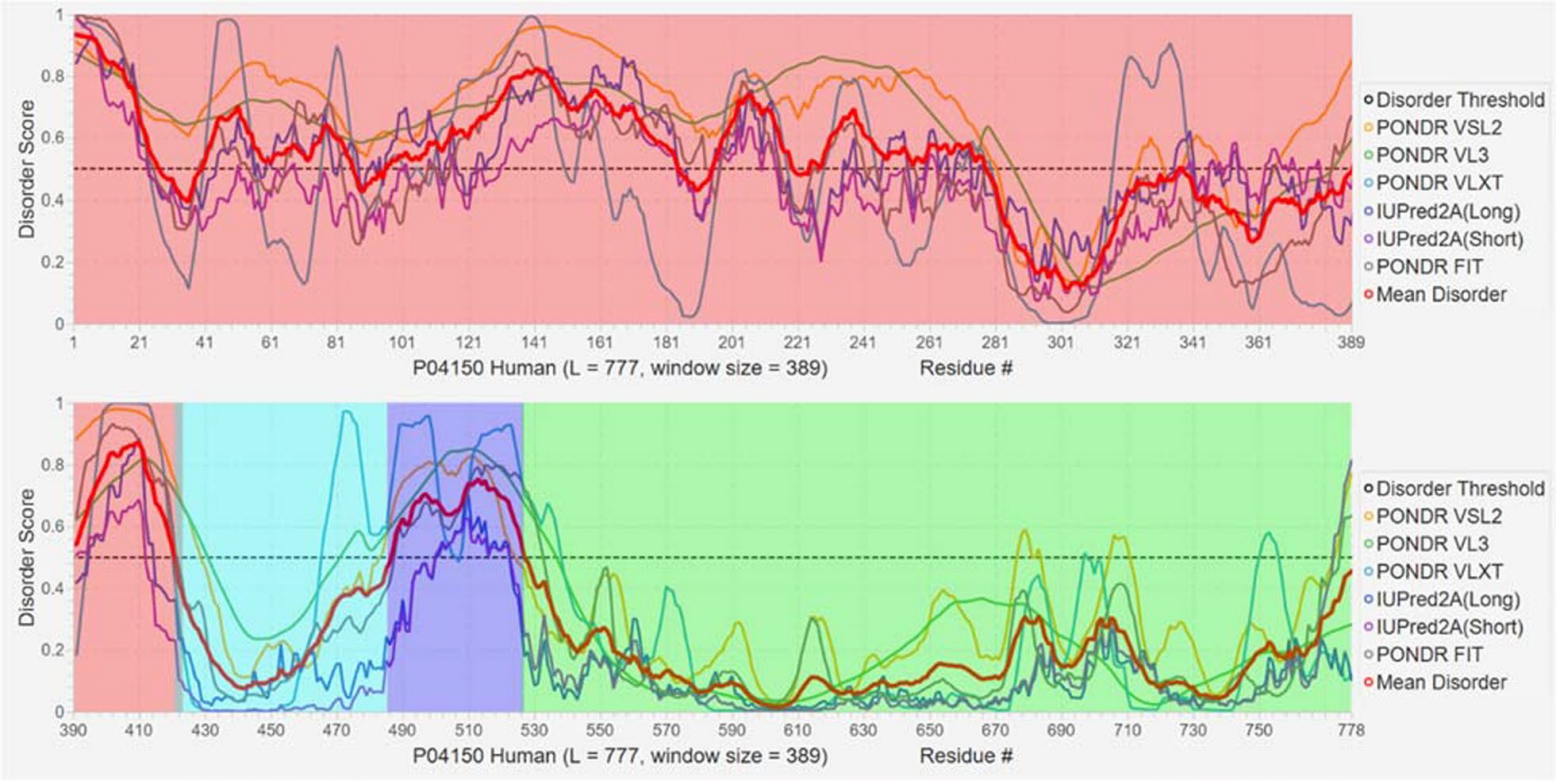
Intrinsic disorder predisposition of human glucocorticoid receptor (UniProt ID: P04150) evaluated by PONDR® VSL2 [179], PONDR® VL3 [180], PONDR® VLXT [6], PONDR® FIT [181], IUPred2A_long and IUPred2A_short [181, 182]. Mean disorder score is shown as well. Positions of the N-terminal domain (residues 1–420), DNA binding domain (residues 420–485), a hinge region (residues 486–527), and ligand binding domain (residues 528–777) are shown as red, cyan, blue and lime shaded areas. Clearly, the N-terminal domain and a hinge region are mostly disordered

*Hormones* Coupling ligand binding to the folding of an intrinsically disordered region expands the range of binding free energies at which allosteric regulation of protein function can occur [176]. One prominent example of this regulatory mechanism is the glucocorticoid receptor, a representative member of the steroid hormone receptor family [176, 177]. The glucocorticoid receptor (GR) consists of a C-terminal ligand binding domain, a central DNA binding domain, and an intrinsically disordered N-terminal domain which is required for the protein to activate transcription (Fig. 2). The ligand binding domain also contains an activation domain, termed AF2. The N-terminal disordered domain is further subdivided into the R and AF1 regions. These regions have different functions, despite the fact that they are disordered, contiguous in the protein sequence, and thermodynamically coupled [176]. AF1 is a transcription activation domain, whereas R is an allosteric repressor of the AF1 domain (reviewed in [178]). Interestingly, the activity of the AF2 transcription activation domain is dependent on ligand binding, whereas AF1 can activate transcription in truncation mutants in which the ligand binding domain is removed, suggesting that the unbound ligand binding domain also inhibits AF1 function in the full-length protein [178].

**Fig. 3:**
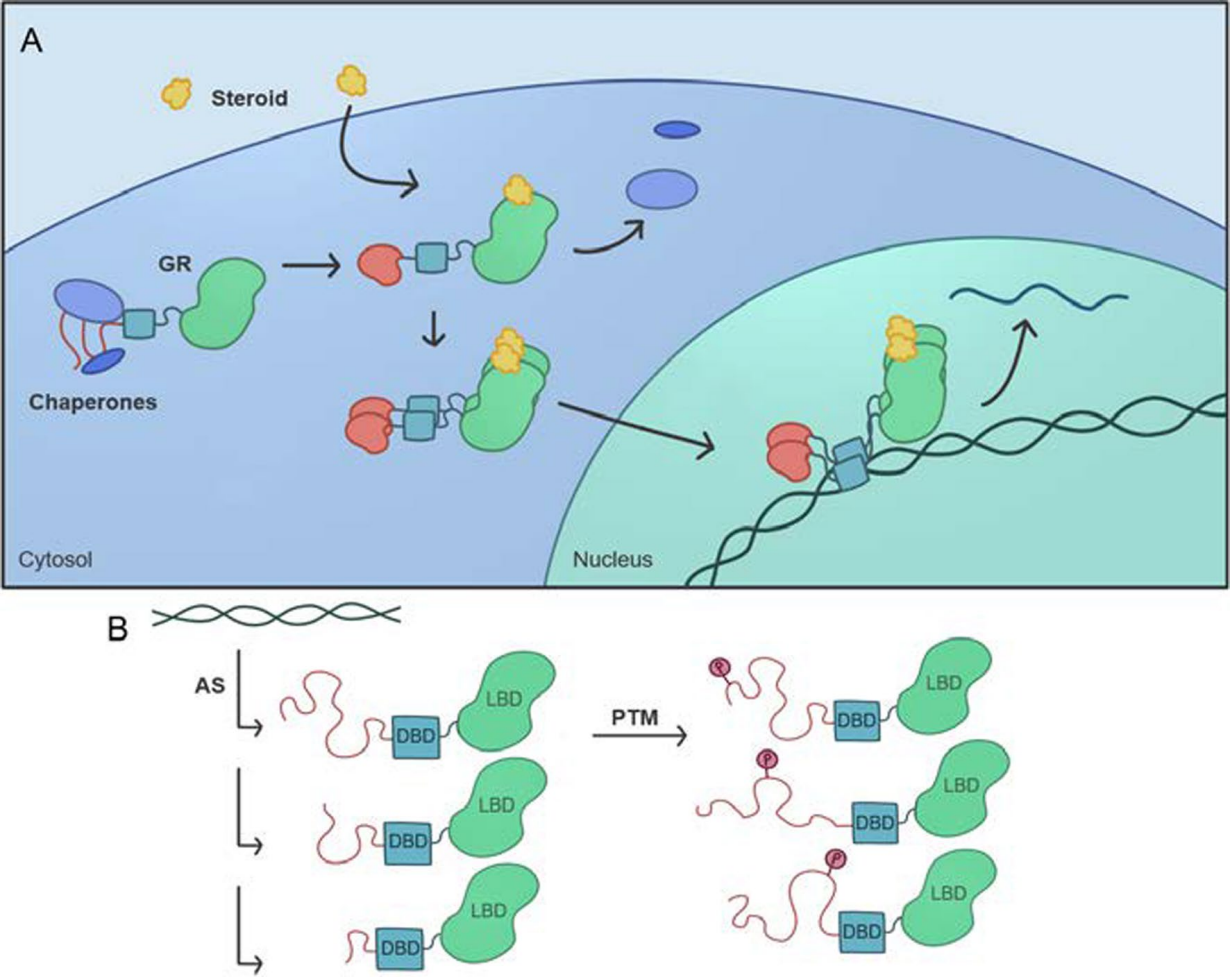
The function of the glucocorticoid receptor is regulated in part by its intrinsically disordered C-terminal tail. **A** The GR tail interacts with chaperones in the cytoplasm in the unliganded state. **B** Alternative splicing and post-translational modifications impact these interactions

The presence of intrinsically disordered regions in a protein allows the allosteric response to be tuned [176]. Prior to activation, the glucocorticoid receptor (GR) resides in the cytosol. In this unliganded state, the N-terminus of the glucocorticoid receptor GR is intrinsically disordered, and interactions with multiple chaperones in the cytosol help GR remain intact and primed for ligand binding [178]. Steroid hormones are able to pass through the membrane to bind their cytosolic receptors, inducing profound structural changes in GR, including folding the N-terminal domain, and release of interactions with cytosolic proteins. GR then translocates to the nucleus where it forms large complexes with co-activator proteins, its target DNA binding sites, and the general transcription apparatus [178] (Fig. 3).

The disorder-to-order transition in the N-terminal domain is regulated by ligand binding and many other factors as well, including interactions with many other proteins, DNA binding, and phosphorylation of the AF1 domain [178, 183]. The multiplicity of activating regulatory mechanisms facilitates AF1’s engagement with co-regulatory proteins and stabilize the final complex. Conversely, disruption of any of these interactions promotes dissolution of the complex, allowing GR to dynamically regulate multiple target DNAs.

Intrinsic disorder in the GR receptor not only enables multiple allosteric regulatory interactions to impact function, but also allows deployment of different surfaces of the protein to enable binding to many different sets of macromolecules, and regulation of these interactions via mRNA splicing and phosphorylation [178]. In addition, combinations of alternative translation initiation and alternative mRNA splicing result in the production of multiple glucocorticoid receptor isoforms from one gene [184]. These isoforms are able to regulate different genes [177]. Furthermore, the use of alternative translation start sites truncates the repressive R region in the disordered N-terminal domain. From our view, alternative splicing and alternative start sites similarly enable one gene to yield multiple transcripts and so are equivalent in this regard [38]. Many additional splicing isoforms and PTM variants have been recently discovered and an associated database has been constructed [185]. The various isoforms exhibit distinctive tissue distribution patterns and altered transcriptional regulatory profiles. Phosphorylation and the binding of additional proteins are discussed above as significant regulators of GR receptors, but these receptors are also regulated by other types of PTMs [186], including ubiquitination, phosphorylation, and sumoylation. These variations likely contribute to the complexity glucocorticoid signaling and help to determine cell-specific response to glucocorticoids [184, 186]. Thus, the GR receptor is a candidate to be a user of the IDP-AS-PTM toolkit for complex context-dependent (e.g. tissue or cell specific) regulation as discussed previously and above for GPCRs, N-FATs, and Sarc Family kinases [39].

*Lipid*s In addition to forming membranes and storing energy, lipids can also activate or regulate cell signaling. Lipid-activated cell signaling also relies on IDPs/IDRs. For example, the Phosphatase and Tensin homolog (PTEN) protein inhibits signaling via the PI3K/AKT/mTOR proliferative pathway, in which phospholipids act as a second messenger. PI3 Kinase bound to an activated receptor phosphorylates PI(4,5)P_2_ to create PI(3,4,5)P_3_, which in turn activates cell proliferation and survival via AKT and promotes cytoskeletal changes via Rac/Rho/cdc42. PTEN dephosphorylates PI(3,4,5)P_3_ to inhibit signaling and thus proliferation. Consequently, PTEN is not only a powerful tumor suppressor, but also a prognostic marker that predicts response in many human cancers [187]. Inactive PTEN exists in a cytoplasmic pool and lacks substrate access [188]. Recruitment and the extent of activation of membrane-associated PTEN depends on the composition, and thus the surface characteristics such as charge, of the membrane (reviewed in [187]). Interestingly, surface charge can also be modified by signaling via phospholipase C [188].

The PTEN protein contains an N-terminal PI(4,5)P_2_ binding site, and a structured catalytic domain followed by an intrinsically disordered auto-inhibitory C-terminal tail which culminates in a PDZ binding domain [188, 189]. The tail contains two groups of serine/threonine residues which can be phosphorylated by kinases such as CK2 and GSK3. Phosphorylation of most of these amino acids forces PTEN into a closed, more stable, inactive conformation, in which membrane association, PDZ binding, ubiquitination, and degradation are all suppressed [188, 190]. Phosphorylation of T366 appears to counter the impact of phosphorylation of the other residues [191]. The disordered tail is also modified via ubiquitination as part of protein degradation, and by acetylation, sumoylation, and S-nitrosylation [192]. Interactions with other proteins via the PDZ binding domain or other regions of the protein both enhances PTEN stability and diversifies its function [188, 192].

Multiple alternate translation and splicing start sites in the PTEN gene creates many versions of the protein, most notably PTEN-L, which contains an extra 173 amino acids on the N-terminus [187, 192, 193]. This region is also primarily disordered and post-translationally modified [192]. Signal peptides within this region facilitate passage into and out of cells and organelles [192–194]. Thus, it is not surprising that PTEN and PTEN-L exhibit different substrate specificities and mechanisms of membrane binding [195]. Again, this protein appears to take advantage of the previously described IDP-AS-PTM Toolkit [39].

*Proteins* Signaling pathways activated by proteins often are regulated by IDPs/IDRs in multiple steps of the pathway. In canonical cell signaling, an extracellular ligand is recognized by a membrane protein which transmits the signal, typically through phosphorylation through a series of cytoplasmic/nuclear proteins, culminating in the regulation of a transcription factor which alters transcription of specific genes. The use of proteins as the signal provides extra regulatory opportunities through modulating signal production (transcription and translation), signaling transport and availability via binding to extracellular matrix proteins, and signal activity via PTMs.

There are many categories of proteins that serve as cell signals. For example, cytokines are small secreted immunomodulatory protein signals. Osteopontin is a multifunctional cytokine with key roles in inflammation, cell viability, and tissue repair, which also functions as a bone matrix protein that mediates osteoclast adhesion [196, 197]. Through these functions, osteopontin is also involved in cardiovascular diseases, cancer, diabetes, and formation of kidney stones [196, 197]. Unliganded osteopontin interconverts between extended, random coil-like conformations as well as a collapsed, cooperatively folded state capable of generating sigmoidal structural denaturation curves [198]. These results suggest that interactions of other proteins with osteopontin generally occur via conformational selection [198].

While cytokines regulate the immune system, growth factors are protein signals that primarily target other types of cells. Vascular Endothelial Growth Factor (VEGF) regulates angiogenesis, and thus plays significant roles in animal development, wound healing, and carcinogenesis. The *vegf* mRNA is alternatively spliced to produce a family of protein isoforms with varying affinities for different VEGF receptors [199]. Active VEGF-A forms a structured dimer with disordered N-terminal and C-terminal tails. Many splice variants of *vegf-a* alter the lengths of these tails (e.g. VEGF_165_ versus VEGF_143_). The VEGF “B” splice variants are generated by use of an alternate 3’ acceptor site for exon 8, lengthening the intrinsically disordered C-terminal tail by an additional 6 amino acids (e.g., VEGF_165_ versus VEGF_165_b) [36, 200]. This addition reverses the function of VEGF: while VEGF_165_ is a potent stimulator of angiogenesis, VEGF_165_b binds VEGF receptors but fails to activate them in a robust, sustained manner. By ineffectively occupying a binding site on the receptor, the VEGF_XXX_b isoforms inhibit the function of the VEGF_XXX_ variants [36, 200]. Inclusion of these extra amino acids also alters the ability of the protein to stimulate proliferation and invasion of non-small cell lung carcinoma cells. Indeed, the ratio of VEGF165b/VEGF165 corelates with lymph node metastases [201].

*Multiple chemical stimuli* Some signaling pathways are capable of responding to a variety of chemical stimuli. For example, the GPCR-G protein signaling system is a complex machine responsible for the recognition of a wide variety of extracellular signals and controls various cellular responses to these signals by triggering the numerous intracellular signaling cascades. The complexity of this machinery is determined by the multitude of the members of the GPCR family (in humans, there are more than 850 different GPCRs [202–205]) that are capable of being recognized and activated by more than one thousand natural and artificial extracellular ligands, ranging from photons to amines, lipids, nucleotides, organic odorants, peptides, and proteins [30, 204]. At the next step, a cytoplasmic domain of an activated GPCR interacts with one of the intracellularly located guanine nucleotide-binding proteins (G proteins), which are heterotrimers composed of α, β, and γ subunits that can control different cellular pathways [206–210]. In humans, there are 23 Gα, 6 Gβ, and 12 Gγ subunits that can be assembled into numerous different heterotrimers [211]. Furthermore, cells contain ~ 40 of the regulator of G signaling (RGS) proteins (which are G protein effectors, modulators, and scaffold proteins) that are capable of interaction with various Gαβγ heterotrimers or their dissociated subunits [212]. All this indicates that the combinatorics of the GPCR-G protein system is gigantic, which can serve as one side of the mechanics of the multitude of corresponding signaling pathways. Recently, based on the comprehensive bioinformatics analysis of human GPCRs and G proteins supported, at least in part, by experimental evidence, it was concluded that intrinsic disorder and associated structural plasticity are crucial for this signaling system [213]. In fact, human GPCRs and G proteins represent dynamic conformational ensembles containing multiple IDPRs and numerous PTMs and MoRFs, and the entire mode of action of these proteins is based on the recognition of a signal followed by conformational change needed for recognition of another partner that is crucial for the downstream transmission of the signal [213]. Therefore, multifunctionality of GPCRs and G proteins, which is required for recognition of a wide variety of extracellular signals and for transmission of this extracellular information for triggering a multitude of the intracellular pathways, is determined by the presence of intrinsic disorder. In other words, this intrinsic disorder-based multifunctionality of the GPCR-G protein signaling system represents an important illustration of the structure–function continuum concept applied to cellular signaling [213]. Finally, as mentioned above, the GPCR molecule is another candidate for taking advantage of the IDP-AS-PTM Toolkit [39].

*Environmental conditions as signals* Unlike many structured protein domains, the function of IDPs/IDRs can persist in extreme environmental conditions. This trait allows IDPs to reliably sense extreme conditions and instigate responsive signaling pathways [26]. The prominent roles that IDPs/IDRs play in responding to light, mechanical forces, pH, redox potential, and drought/salt concentration are discussed below.

*Light* Plants must sense and adapt to light in order to optimize energy production, to limit photodamage, and to set/maintain their circadian clock. The UVR8 photoreceptor in plants is crucial for generating photomorphogenic and protective responses to UV light [162]. The UVR8 protein includes intrinsically disordered N- and C-terminal tails which regulate protein activity [162]. UVR8 is partially inactivated by dimerization, producing an equilibrium between an inactive compact dimer and an active extended monomer. Photoexcitation of UVR8 triggers dimer dissociation and enables the extended C-terminal tail to bind COP1, which regulates light signaling in plants, and propagates the signal [162]. Conversely, active monomers also bind RUP proteins, which inhibit UVR8 signaling.

To avoid harmful light exposure, blue light is used by free-swimming bacteria to modulate both the length and directionality of their run [214]. To this end, several specific proteins are used as blue-light photoreceptors. An illustrative example of action of such photoreceptors is given by photoactive yellow protein (PYP) from a motile, alkalophilic and halophilic bacterium *Ectothiorhodospira halophila*. This water-soluble ~ 14 kDa protein contains a thioester-linked p-coumaric acid cofactor and acts as the photosensor [215–218]. Upon light excitation, *trans/cis* isomerization of a double-bond in the chromophore triggers a cycle of structural events yielding a long-lived, blue-shifted intermediate (known as pB) with a life-time on the order of 1 s [216, 219]. High-resolution solution NMR spectroscopy demonstrated that this long-lived pB intermediate is characterized by a noticeable level of disorder and exists as an ensemble of multiple conformers interconverting on a millisecond time scale [220]. Although these light-induced structural perturbations affected almost the entire molecule, the ordered structure of PYP is restored once pB converted back to its ground state (pG). This cycle of light-induced unfolding and dark-promoted refolding has been proposed to regulate protein function, with the disordered pB state being able to bind partner molecules, allowing the swimming bacterium to operate the directional switch that protects it from harmful light exposure [220].

*Redox potential* The conditionally disordered chloroplast protein of 12 kDa (CP12), found in the chloroplasts of photosynthetic organisms such as plants, cyanobacteria, algae, and cyanophages. CP12 regulates the Calvin-Benson-Bassham cycle, which is a series of redox reactions that converts carbon dioxide into glucose [26]. The extent of disorder, and thus the activity, of CP12 is determined by redox conditions, although CP12 remains highly mobile in both the oxidized and reduced states. In dark or oxidizing conditions, CP12 forms limited, marginally stable structure and 2 disulfide bonds which are required to bind and inactivate two enzymes that participate in the Calvin-Benson-Bassham cycle (glyceraldehyde-3-phosphate dehydrogenase (GAPDH) and phorphoribulokinase (PRK)). In light/reducing conditions, the disulfides bonds break and the CP12-GAPDH-PRK ternary complex dissociates, re-activating the enzymes and thus carbon fixation.

*Mechanical forces* Many cellular processes that are regulated by chemical stimuli, such as proliferation, differentiation, motility, and survival, are also influenced by the mechanical properties of the substrate supporting the cells [221]. Mechanosensing/mechanotransduction induces cellular responses to compression, tensile stress, shear stress, and hydrostatic pressure. Alterations in tissue stiffness are associated with many diseases, including cardiovascular disease, muscular dystrophy, and cancer [222]. Mechanical stress is transmitted between cells via cell–cell adhesion adherens junctions composed of the ABE complex (alpha-catenin, beta-catenin, and epithelial cadherin cytoplasmic domain) [223]. The ABE complex is flexible and pliable, and thus adopts a wide variety of structures [223]. This structural versatility arises from protein-domain motions in α and β catenin, and is thought to provide reversibility and sensitivity to stress sensing [223].

In a second example, the mouse protein CasSD includes an intracellular, proline-rich disordered domain. In the absence of mechanical stretching forces, this region formed polyproline II helices hypothesized to bind LIM domain proteins, thus protecting CasSD from phosphorylation. Application of mechanical stretch has been proposed to unfold the PPII conformation, precluding LIM protein binding, thus enabling CasSD phosphorylation and signal propagation [224]. Mechanical stretching similarly unfolds talin and other mechanosensitive proteins, thus exposing cryptic binding sites [225, 226].

*pH* The low pH of the mammalian stomach is one of the natural barriers to protect the organism from microbial infection. Enteric bacteria, such as *E. coli*, have adapted to sense, respond to, and survive in acidic environments. Sudden immersion in acid requires an immediate adaptive response. Thus, the extended signaling pathways used in non-life-threatening situations in other organisms are reduced to single protein sense-and-respond units to detect acid in bacteria. For instance, the CadC protein is a pH-responsive one-component signaling system composed of an N-terminal winged helix-turn-helix DNA binding domain, followed by a 50 amino acid intrinsically disordered region linking to a single transmembrane helix and ending in a C-terminal pH sensing domain which extends into the periplasm [161]. Intriguingly, the disordered linker is required to transduce the pH-dependent status of the periplasmic sensing domain to the DNA binding domain via dimerization. Likewise, the *E. coli* periplasmic protein HdeA behaves as an acid-inducible chaperone [227]. At neutral pH, HdeA is folded and inactive. Exposure to acidic conditions unfolds HdeA, allowing it to bind its substrate proteins.

*Hydration* Dehydrins are a family of intrinsically disordered proteins that act as effectors whose expression is induced by signaling pathways that sense abiotic stresses, such as cold or draught. Dehydrins protect plants from dehydration and from cold [228]. Although the impact of cold in plants is amplified by dehydration, Dehydrins can protect both protein activity and membrane structure [228]. Hydrophobic amino acids are necessary for these activities [229]. Dehydrin proteins are categorized by the presence of three conserved motifs—the K-, Y-, and S-segments, which are enriched in lysine, tyrosine, and serine respectively. Not all proteins contain all three motifs, although more than one copy of any motif may be present. Specific dehydrins may also contain additional motifs that impart additional functions (e.g., a poly-histidine region confers metal binding and self-dimerization) [228]. In vitro, interactions with metal ions, other proteins, and sodium dodecyl sulfate induce the formation of limited structure for some dehydrins [228, 229], which can be transiently stable in vivo [230].

*Heat* The dehydrin protein family discussed above may also protect from heat. The *Arabidopsis thaliana* dehydrin Early Response to Dehydration 14 (ERD14) can increase the viability of *E. coli* subjected to 15 min at 50 °C [230]. This protection relies on K- and H-segments which form stable helices upon binding to partner proteins in vivo. Multivalent binding of one ERD14 to difference surfaces of a single partner protein holds different regions of the same protein in close proximity. Conversely, multiple ERD14 proteins that each bind multiple partners both sequester exposed hydrophobic residues and prevent partner proteins from approaching one another, which would otherwise lead to aggregation.

## IDRS/IDPS are found in every category of cell signaling pathways

Based on the source of the signal and the relative location of the responding cell, cell signaling is divided into autocrine, juxtacrine, intracrine, paracrine, and endocrine pathways. Despite the large variety of signal transmission mechanisms used, IDRs/IDPs act as crucial components in each of these different categories.

Intracrine signaling self-regulates by producing hormones that bind intracellular receptors. Thus the cell stimulates itself because the signal, and hence the signaling cascade, never crosses the cell membrane. The nature of intracrines can vary: structurally diverse hormones (e.g., steroid hormones (which are mostly synthesized from cholesterol), growth factors, DNA-binding proteins, and enzymes all can have intracrine activity [213]. Furthermore, several protein/peptide hormones might act as intracrines as well, with the most notable example (in the light of the ongoing COVID-19 pandemic) being peptides of the renin–angiotensin system, such as angiotensin II and angiotensin, conversion between which is controlled by the angiotensin-converting enzyme 2 (ACE2), which also serves as the main entry point into cells for some coronaviruses including SARS-CoV-2 [231]. In the intracrine signaling pathways, the corresponding intracrines are recognized by and act through the specific intracellular receptors, which are often of nuclear or cytoplasmic origin. In the case of steroid hormones, the most studied intracellular receptors are the members of the nuclear receptor subfamily 3 (NR3) that include receptors for estrogen (group NR3A) [232] and receptors for 3-ketosteroids (group NR3C) [233], which first form a complex with the hormone binding estrogen receptors (ERs) and then activate transcriptional processes and/or signaling events that result in the control of the gene expression. There are two ERs in humans, ERα (595 residues) and ERβ (530 residues), which can exert their regulatory functions via genomic [234] and non-genomic estrogen-mediated signaling events [235]. In direct genomic signaling, ERα and ERβ act as ligand-activated transcription factors. Here, hormone binding triggers conformational changes and dimerization of the receptor leading to the translocation of the complex to the nucleus, where it binds to the chromatin at specific DNA sequences known as estrogen response elements (EREs), which are present in many gene promoters [236]. However, ~ 35% of genes targeted by estrogen lack ERE-like sequences [237, 238], and the corresponding genes are expressed via “indirect genomic signaling” or “transcriptional cross-talk”, where the ER complexes operate via interactions with other transcription factors [239]. Furthermore, ERs can be activated in the absence of estrogen by either phosphorylation at specific residues [240] or by interaction with co-regulators, co-activators and co-repressors, that can enhance or decrease transcriptional activity of ERs, respectively [241, 242] and which are regulated by various PTMs themselves [243]. Therefore, ERs act as multifunctional proteins capable of (a) interaction with small molecules-ligands; (b) undergoing conformational changes triggered by hormone binding; (c) oligomerization; (d) translocation to nucleus; (e) interaction with DNA; (f) interaction with other transcription factors; (g) interaction with various co-activators; and (h) undergoing various PTMs. Collectively, these abilities clearly indicate that the activity of ERs relies on intrinsic disorder [17]. In line with this hypothesis, there are several studies showing that ERα contains an intrinsically disordered transactivation domain (AF1) in its N terminus [244], activity of which is regulated by phosphorylation and associated phosphorylation-coupled proline isomerization [245, 246].

Autocrine signaling occurs when a cell is stimulated by a signal that was produced and secreted by that same cell. Autocrine brain-derived neurotrophic factor (BDNF) has been implicated in the structural and functional plasticity of dendritic spines [247]. BDNF also regulates neuronal plasticity, including structural long-term potentiation, an aspect of learning. The BDNF proprotein must be cleaved to form an intrinsically disordered N-terminal prodomain, and a structured C-terminal protein which dimerizes to form mature BDNF (mBDNF), both of which are secreted and have activity [248]. The autocrine mBDNF promotes neuronal survival, growth, and development. The cleaved prodomain, proBDNF, binds other proteins to promote cell death, dendritic remodeling and long-term depression. Interestingly, a mutation in the prodomain, V66M, is present 20% of humans and increases the occurrence of anxiety disorders, depression, memory deficits, and impairs recovery after traumatic brain injury (reviewed in [248]). The wild type and mutant prodomain exhibit few differences in structure or protein interactions. However, binding by Zn^2+^, which is present outside neurons, creates distinct conformations and dramatically alters prodomain oligomerization.

Juxtacrine signaling requires contact between cells, allowing a ligand on one cell surface to bind a receptor on an adjacent cell. In metazoans, Notch signaling plays key roles in early stages of embryonic development, as well as development of the cardiovascular system, the central nervous system, and the respiratory system, among others, and has well-defined roles in cancer progression [249, 250]. Notch and its ligands are all transmembrane proteins, in which the extracellular domains mediate the inter-protein interactions that activate the pathway. Upon ligand binding, the Notch intracellular domain, which includes a 111-amino acid intrinsically disordered region, binds the transcription factor CSL (an acronym of human and mouse CBF1/RBPJ-κ, *Drosophila* Suppressor of Hairless, and *C. elegans* Lag-1), the coactivator Mastermind, and Notch target DNA [251]. Within the Notch intracellular domain, both the N-terminus of the disordered region and the ankyrin repeat region bind distant sites on CSL, with the remainder of the disorder region linking the two interaction regions. The pattern of positive and negative charges within this linker region are thought to mediate additional interactions to stabilize the complex [252].

A specialized type of juxtacrine signaling is enabled by gap junctions. Gap junctions allow ions and small metabolites to exchange between adjacent cells, and are formed by two connexin proteins, one provided by each cell [253]. The intrinsically disordered C-terminal domain of connexins (148 amino acids) determines whether the channel is open or closed. Phosphorylation in this region regulates protein interaction, channel assembly, channel degradation, and metabolic and electrical coupling, and thus controls trafficking through the channel [253]. Different connexin proteins are expressed in different tissues, and respond differently to phosphorylation. For instance, Connexin 32 is expressed in the liver and brain, whereas Connexin 43 is produced in cardiac muscle [253, 254]. Phosphorylation of the C-terminal domain of Connexin 43 inhibits channel function, whereas phosphorylation of Connexin 32 stimulates channel function [254].

Paracrine signaling involves the release of diffusible chemical signals to communicate between nearby cells in which cell–cell contact is not required. One prominent example is neurotransmission. Glutamate is the primary neurotransmitter for excitatory stimulation. Signaling via glutamate is a critical component of long-term potentiation and long-term depression, which alter the strength of downstream signaling in response to glutamine binding to receptor. These adaptations are crucial events in learning and the formation of memory [169]. Glutamate binds both ionotropic glutamine receptors and metabotropic glutamine receptors. Both have long cytoplasmic C-terminal intrinsically disordered domains (CTDs) [169, 255]. The CTDs of both receptors are alternatively spliced, and post-translationally modified (phosphorylation and SUMOylation for metabotropic receptors, phosphorylation and palmitoylation for ionotropic receptors) [169, 255]. These modifications diversify the intracellular sites available for protein interactions, allowing different complexes to be formed and altering the transmitted signal [169, 255].

In endocrine signaling, endocrine cells produce signals that target distant cells in the body. Most of the intrinsically disordered proteins with well-studied roles in signaling operate in endocrine signaling pathways. The Wnt signaling pathway exemplifies how intrinsic disorder can play multiple roles in a single pathway (Fig. 2). A mouse oncoprotein signal (Int-1) and a *Drosophila* body-plan-controlling developmental protein (Winged) were identified as homologues, leading to the portmanteau Wnt as the family name for these proteins [256]. The Wnt family signaling proteins are both glycosylated and palmitoylated and are universal across multicellular members of the animal kingdom but absent in single cell members, with a few paralogues in sponges and with 19 paralogues in humans [257, 258, and The Wnt Homepage (stanford.edu)]. Humans also have 10 members of the Frizzled (Fz) protein family, which serve as Wnt receptors [258]. While some biological processes may integrate signals propagated by multiple different Wnt-Fz complexes, some Wnt-Fz complexes drive opposing biological responses, indicating that Wnt-Fz interactions must have the capacity for selectivity. This selectivity can be achieved by formation of larger Wnt-receptor complexes, in which Fz interaction is mediated by conserved Wnt residues, and divergent, intrinsically disordered regions of the same Wnt bind additional receptors, such as Reck [258].

Prior to Wnt signaling, the transcription factor β-catenin is maintained at low levels by the β-catenin destruction complex, which is an assembly of the 852 residue mostly disordered scaffold protein axin, the 2,843 residue massively disordered protein adenomatous polyposis coli (APC), and the three mostly structured proteins β-catenin, casein kinase Ia (CKI-a), and glycogen synthetase kinase 3b (GSK3b). The two kinases phosphorylate residues within a long IDR at β-catenin’s amino terminus [259, 260]. Indeed, most protein phosphorylation occur in IDRs [41]. The phosphorylation modifications signal β-catenin’s subsequent ubiquitination, which also occurs primarily within IDRs [261]. Next, the β-catenin proteins undergo proteasomal destruction [259, 260], which is greatly accelerated by the presence of IDRs in addition to the bound ubiquitin signal [262].

The two kinases and β-catenin bind to Axin’s long intrinsically disordered region, thereby connecting structured proteins with flexible linkers. Random movements of the bound proteins enabled by the flexible linkers bring about frequent kinase-substrate collisions, which, in turn, lead to efficient phosphorylation. Thus, the destruction complex works by random movements of a ‘‘stochastic machine,’’ not by cooperative conformational changes [259].

Laboratory experiments show that a 55 residue segment of axin containing binding sites for both β-catenin and GSK3b has the biophysical properties of an unstructured protein [263], which is in agreement with the predictions of disorder on this protein. Furthermore, addition of this disordered segment to solutions of β-catenin and GSK3b greatly accelerates the rate of phosphorylation of β-catenin by GSK3b. If too much of this disordered peptide is added, the elevated phosphorylation rate decreases, likely because, in the presence too much peptide, many peptides bind either GSL3b or β-catenin but not both proteins. Finally, if shorter fragments containing either one of the two binding sites are added to solutions of β-catenin and GSK3b, no rate acceleration is observed. Overall, these data show that the phosphorylation rate enhancement occurs by the binding of both the kinase and the substrate to a single flexible tether and not by activation resulting from the binding of axin to the enzyme or the substrate [263]. These experiments provide strong experimental validation of the stochastic machine model [259].

Upon encountering the target cell, Wnt binds to two co-receptors, the seven transmembrane helix protein Frizzled (Fzd) and the single pass lipo-related-receptor protein 5/6 (LRP5/6). This binding promotes recruitment of the scaffolding protein disheveled (Dvl), which results in the phosphorylation of the cytoplasmic domain of LRP5/6, a domain whose disorder [264, 265] reflects disorder in the cytoplasmic domains of other single pass membrane proteins [266] and like most other segments that undergo phosphorylation [41, 265]. The resultant molecular complex Wnt-Fzd-LRP5/6-Dvl forms a structural region for Axin interaction that disrupts Axin-mediated phosphorylation/degradation of the transcriptional co-activator β-catenin, thereby allowing it to stabilize and accumulate in the nucleus where it activates the expression of multiple Wnt-dependent genes.

Due to its prominent physiological function, the Wnt/β-catenin signaling must be strictly controlled because its dysregulation, which is caused by different stimuli and also by many different mutations that lead to alterations in cell proliferation, apoptosis, inflammation-associated cancer or alterations in stem cell proliferation or self-renewal, for both embryonic and various types of adult stem cells [257].

## IDRS/IDPS are found in every step of cell signaling pathways

**Fig. 4:**
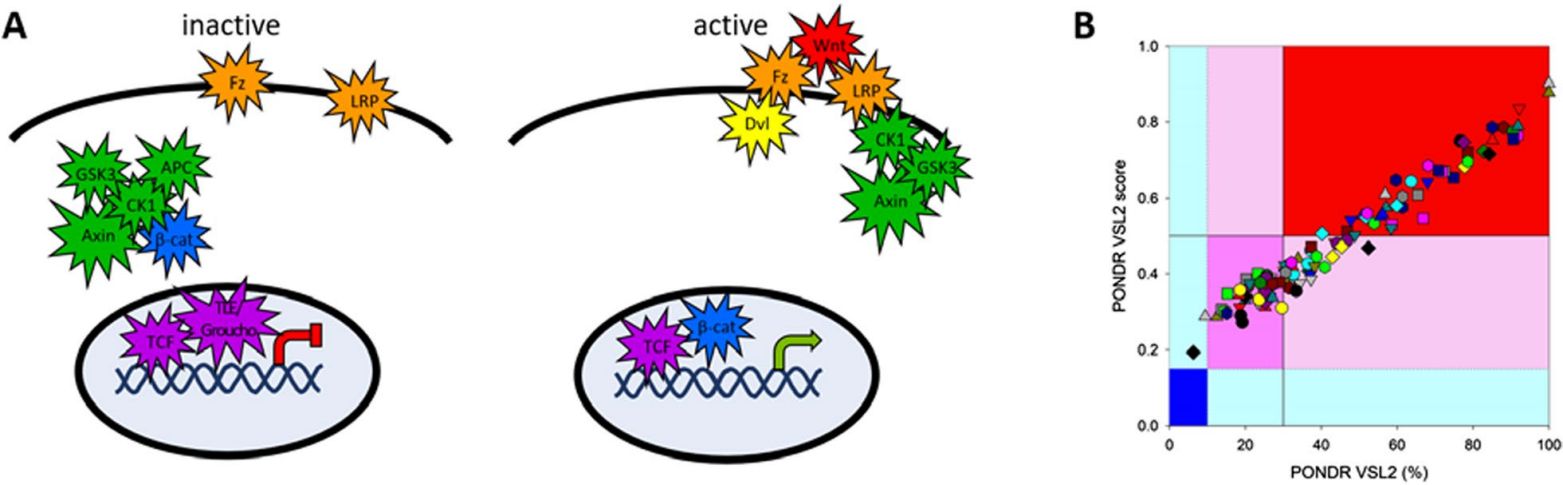
Disorder can occur at any step of the Wnt cell signaling pathway. **A** A schematic of signaling components in the core canonical Wnt signaling pathway, showing the inactive state on the left and the active state on the right. The cell membrane is indicated by an arc and the nucleus by a light blue oval. Wnt signaling is able to control many processes by employing different variants of many proteins involved in Wnt signaling, which exist due to gene duplication, alternative splicing, and PTMs [258]. Consequently, a protein was marked as disordered (using a starburst) if the sequence off any variant, not post-translationally modified, was identified as intrinsically disordered in the literature. Wnt [258], Fz [268], LRP [268], Dsh/Dvl [264, 265], APC, CK1, Axin, GSK3 [269], β-catenin [269, 270], TCF/LEF [271], Groucho [272] all can include intrinsic disorder. **B** Analysis of 117 proteins involved in Wnt signaling based on disorder score and percent of disordered residues. Large values of each parameter indicate increasing disorder. Color blocks indicate regions in which are mostly ordered (blue and light blue), moderately disordered (pink), or mostly disordered (red). If the two parameters agree, the corresponding part of background is dark (blue or pink), whereas light blue and light pink reflect areas in which only one of these criteria applies. It is noteworthy that no Wnt pathway proteins are very structured (dark blue) and only two proteins can be considered mostly disordered. The remaining 115 proteins are either moderately disordered or highly disordered

The sections above highlight the different structures of cell signaling pathways. Intrinsic disorder may be present, and provide regulatory opportunities, for any of the following steps: ligand production, ligand activity, ligand bioavailability, receptor structure, intracellular transmission, termination/intracellular trafficking, and effector proteins (Fig. 4). Indeed, in addition to Wnt signaling, ten other pathways associated with development of multicellular metazoans, including pathways also associated with cancer, or also associated with stem cell proliferation were tested for their utilization of IDRs. Like Wnt, all ten additional developmental pathways also extensively used proteins containing IDRs [267].

*Ligand production* The production of many signaling molecules is highly regulated at the level of gene transcription. Furthermore, the transcription factors involved are often regulated by other signaling pathways (Fig. 4). Since intrinsic disordered regions are highly prevalent in transcription factors [273–277], intrinsic disorder is a major factor in regulating the production of cell signals.

*Ligand activity/bioavailability* The bioavailability of protein ligands is determined by highly regulated interactions with proteoglycans, which are ubiquitous components of the extracellular matrix. Heparin is a glycosaminoglycan in which disaccharide units may be sulphated [278]. Heparan sulfate proteoglycans (HSPGs) consist of a protein core with chains of heparan sulfate covalently bound. Most cells express at least one HSPG. Heparin binds > 400 proteins, including many involved in cell signaling [279]. Examples include growth factors such as FGF, VEGF, and HGF, EGF, and pro-inflammatory cytokines such as IL-8 [278, 280]. GFs bound to HSPGs are sequestered and thus not active [280]. Cleavage of heparan sulfate by Heparanase releases these signaling proteins [280]. Heparanase levels are regulated to control signaling and are elevated in tumorigenesis, metastasis, and angiogenesis [280]. Likewise, the affinity of cell signals for heparin is a major determinant of signaling strength. Proteins bind heparin via intrinsically disordered sequences rich in lysine and arginine [259]. The affinity of growth factors/cytokines for heparin correlates with the percentage of disordered residues in heparin-binding sites [259].

*Receptor structure* Transmembrane receptors transduce the signal generated by ligand binding across the membrane. Many receptors require intrinsically disordered cytoplasmic tails to function properly [169, 281–283]. In a common strategy, conformational changes in the receptor triggered by ligand binding promote release of the cytoplasmic tail from association with the membrane. Once free, disordered tails engage in the protein–protein interactions required to propagate the signal. For the Epidermal Growth Factor Receptor (EGFR), this phenomenon is observed in the juxtamembrane region, which links the transmembrane α-helix with the tyrosine kinase domain. Prior to ligand binding, both the monomeric and inactive dimeric conformations of EGFR allow basic residues in the juxtamembrane region to bind the membrane. Upon ligand binding, the transmembrane helix re-arranges and EGFR forms active dimers [284, 285]. In the active dimer, the lipid bilayer releases the two juxtamembrane regions, enabling them to form antiparallel helices. This conformational change promotes autophosphorylation, and hence activation, of the two tyrosine kinase domains [281]. This arrangement can be regulated by altering the affinity of the juxtamembrane region for the membrane: PIP_2_ binds the juxtamembrane region to facilitate dimerization, whereas T654 phosphorylation decreases membrane affinity and thus activation [281, 286]. Furthermore, oncogenic mutations that stabilize the juxtamembrane region cause EGFR to be constitutively active [287].

IDPs/IDRs are particularly enriched in signaling proteins associated with membranes. Because the presence of intrinsic disorder provides unique opportunities for interactions with membranes (reviewed in detail by Cornish et al. [281]), it is perhaps not surprising that 15% of all disordered proteins bind lipid [288, 289]. The enrichment of positively charged amino acids within disordered regions enables electrostatic interactions with lipid head groups, which can induce membrane curvature [281]. Conversely, membrane curvature can reduce the motion, and hence conformational entropy, of disordered regions, allowing these proteins to act as curvature sensors. Disorder would expose any hydrophobic side chains, allowing their insertion into the membrane [281]. When receptors, scaffolds, and intracellular mediators of cell signaling pathways serve as protein interaction hubs, the membrane increases their effective concentration and restricts diffusion to two dimensions, thus increasing the probability of protein interactions. The presence of the membrane as a physical barrier can sterically prevent non-productive interactions from forming. Furthermore, the orientation of one protein to the membrane can expose or hide protein binding sites and thus regulate signal progression through the pathway [290].

Integrins not only mediate two-way communication between the cell interior and the extracellular matrix, but they also regulate ion channels, growth factor receptors, and the activity of cytoplasmic kinases [291]. These regulatory interactions allow integrins to coordinate cytoskeletal structure with growth factor-mediated processes such as cell adhesion, migration, and invasion of the extracellular matrix. The affinity of integrins for their ligands/the extracellular matrix is regulated by their intrinsically disordered cytoplasmic tails. These tails also act as a hub to form and regulate intracellular protein complexes [292–294]. The ability of integrins to bind extracellular ligands is regulated by talin, a cytoplasmic cytoskeletal protein [295–298]. The α-helical propensity, dynamics, and affinity in the β tails of integrins strongly suggest that conformational entropy plays an important role in Talin binding, with a preformed helix binding more readily than a disordered one [299].

**Fig. 5:**
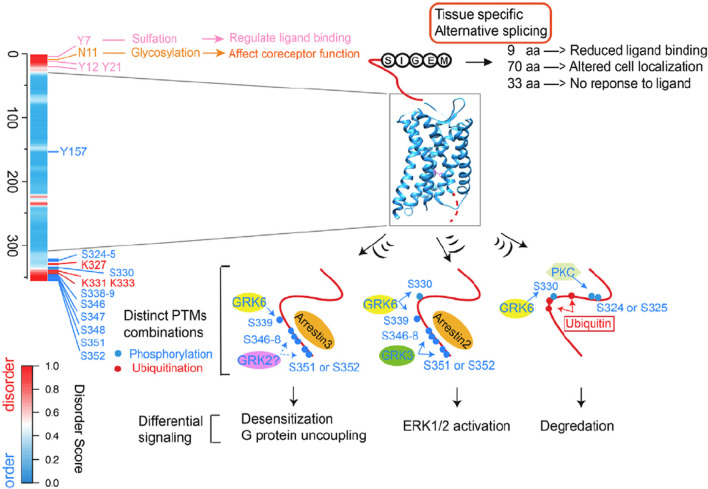
Alternative splicing and PTMs, localized in intrinsically disordered regions, direct differential CXCR4 signaling. Predicted disorder identified by PONDR-FIT is depicted on a heat map (lower left), with red and blue indicating predicted disorder and order, respectively. A crystal structure of the structured regions (28–303 residues, PDB ID: 3OE9) is shown as a blue ribbon. Alternative splicing regulates receptor function by generating three tissue-specific isoforms by replacing the first five residues at the disordered N-terminus with other sequences of varying length. Multiple PTMs regulate different aspects of CXCR4 function: sulfation of Y7, Y12, and Y21 modulates receptor-ligand binding and dimerization [300], and glycosylation of N11 plays a role in masking the coreceptor functional activity [301]. Likewise, phosphorylation of Y157 is required for activation of the G_i_-independent JAK2/STAT3 pathway [302]. Consequently, combinations of C-terminal PTMs are associated with three different biological processes: phosphorylation of S339 in G protein-coupled receptor kinase 6 (GRK6) and possibly GRK2 phosphorylation (two residues from S346-S348 and S351-S352) lead to receptor-arrestin3 binding, G protein uncoupling, and subsequent receptor desensitization. In contrast, phosphorylation of GRK3 (at the same regions as GRK2, but probably different residues), and GRK6 (S330 and S339) result in arrestin2 recruitment and subsequent ERK1/2 activation [303]. In addition, protein kinase C (PKC) and GRK6 phosphorylation (S324 or S325, S330 respectively) initiate degradation modulated by ubiquitination of K327, K331, and K333 [303, 304]. Adapted from Zhou et al. [39]

Similar regulatory mechanisms have been established for G-Protein Coupled Receptors (Fig. 5), which were recently reviewed by Zhou et al. [39].

*Large multi-site docking proteins (LMDs)* leverage the protein binding capacity of intrinsically disordered tails. Many cell signaling pathways require large multi-site docking proteins to transduce signal from the activated receptor to downstream intracellular effectors [305]. Signaling hubs bind many proteins, but are limited to a few interactions at a time. This arrangement can allow response to a single signal to evolve with time or enable one protein to transmit multiple different signals based on the protein interactions formed [281]. Scaffold proteins spatially and temporally regulate cell signaling pathways by binding and sequestering signaling proteins [306]. Thus, LMDs bind to both integrate signals from multiple pathways and coordinate the downstream response [27, 307, 308]. Formation of these higher-order complexes allows amplification and integration of multiple signaling pathways instigated by cytokines, growth factors, and antigen receptors [27, 119, 309]. For instance, disordered hub regions can facilitate engagement of kinases with target proteins [310]. Gab2 is a type of LMD protein that operates as part of many signaling pathways [308, 311] and transmits signals from integrins, receptor tyrosine kinases, cytokine receptors, multi-chain immune recognition receptors, and G protein-coupled receptors, and is required to activate Akt, Ras/Raf, Rac, c-fos, Jak/Stat, Rac, and a host of other intracellular transducers [308, 311, 312]. Upon phosphorylation by protein tyrosine kinases, Gab2 binds both intracellular domains of receptors and many signaling proteins to activate multiple pathways by bringing the necessary factors into proximity [305, 308, 313].

Although most of the 74 kDa Gab2 protein is intrinsically disordered, it does contain a folded PH domain at its N-terminus, which anchors Gab2 to the membrane via interactions with the lipid PIP3 [308]. Gab2 function is critically dependent on binding to Grb2, which physically links Gab2 to the activated receptors [27, 314]. The Gab2-Grb2 interaction illustrates how complexes are organized by the long, disordered tails in the LMD class of proteins. Even though the disordered region of Gab2 is ~ 550 amino acids long, only two short regions (~ 20 amino acids) interact with Grb2, both binding the C-terminal SH3 domain of Grb2 [313, 315]. In isolation and in solution, the interacting regions of Gab2 are mostly disordered, with some residual signatures of extended β and polyproline II conformations [27]. Studies on the similar LMD protein Gab1 demonstrate that allosteric interactions and binding-induced folding are critical for the correct formation of these multiprotein complexes [307].

Proteins that bind to Gab2 often contain an SH2 protein interaction domain [305, 308, 313]. SH2 domains, which were discovered by Tony Pawson and colleagues, are non-catalytic structured domains that bind target sequences containing a phosphorylated tyrosine, and are found in several different multidomain proteins [316]. The many protein interaction domains are each wide-spread and found in multiple proteins, and their associated binding specificities have revolutionized our views of cell signaling [317]. The 14-3-3 proteins and proteins with phospho-tyrosine binding domains also use structure to bind to DBRs located in IDRs. We wondered whether the use of DBRs in IDRs for associating with protein interaction domains is rare or common. A convenient source containing more than 80 well characterized protein interaction domains is the Pawson Lab website (search “The Pawson Lab—Home”, click “domains—map”). So far more than 30 of these protein interaction domains have been shown by published experiments and/or by prediction to bind to DBRs in IDRs, with none so far binding to structured domains (work in progress). We suggest that developing a comprehensive list of protein interaction domains from a given eukaryotic model organism, then determining which ones bind to DBRs located in IDRs and which ones, if any, bind to structured proteins would be a very useful exercise.

Intracellular transmission of signals relies on a series of protein interactions. Many kinases include IDRs, which facilitate the intermolecular interactions critical for the function and specificity of the signaling cascade [318]. For interactions mediated by disordered tails, the disordered character of these regions provides multiple opportunities for regulation that can be applied simultaneously to diversify potential outcomes and refine the cell’s response. For instance, Ras, a p21 GTPase, is activated (1) by receptor tyrosine kinases (2) when bound to GTP and (3) when anchored in the membrane. Once activated, Ras binds its effector proteins, activating signaling cascades that control cell proliferation and survival until GTP hydrolysis switches the signaling off [319]. Although the catalytic domain is highly conserved among Ras family proteins (90–100% identical), the disordered C-terminal hypervariable regions exhibit substantial sequence diversity. Membrane anchoring positions the catalytic domain relative to the membrane and effector binding sites in the tail [320]. The disorder in the tail enables the occurrence of PTMs that add lipids and other groups to the tail, regulating membrane anchoring, domain positioning, auto-inhibition, effector protein binding, and, ultimately, Ras-mediated signaling [320, 321].

The function of K-Ras, a member of the Ras family with a lysine-rich tail, can be affected by alterative mRNA splicing which alters the amino acid sequence of the tail (e.g., generating K-Ras4A and K-Ras4B). K-Ras4B is an oncogenic isoform of Ras, in which GTP hydrolysis, aided by the GAP protein, is sterically obstructed [320]. Consequently, the duration of K-Ras4B activation is longer than the other Ras isoforms. In addition, the effector binding side, which is inaccessible in inactive Ras, is more exposed in this oncogenic Ras isoform. Thus, the disordered tail controls many of the functions of this critical protein, and sequence variations in the tails create a family of Ras proteins that recognize different effectors and have unique activity profiles [319, 322]. Many of the regulatory mechanisms available to IDPs/IDRs are employed by Ras. Ras and its various isoforms and functions are extensively reviewed by Cornish et al. [281].

*Transcription factors* Greater than 90% of transcription factors either contain IDRs or are entirely intrinsically disordered, thus it is not surprising that transcription factors regulated by cell signaling are also likely to include intrinsically disorder [275, 323]. Many transcription factors contain structured DNA binding domains, whereas the domain(s) that regulate transcription activation and repression are unstructured. An example of cell signaling-regulated transcription factors with this organization includes Gli3, a transcription factor regulated by Sonic Hedgehog signaling, which has an intrinsically disordered repression domain embedded with protein interaction sites [324]). Conversely, an example of a signaling-regulated transcription factor with a disordered DNA binding domain is the LEF/TCF protein Lymphoid enhancer-binding factor-1 (LEF-1) [271]. In response to Wnt signaling, LEF-1 bound to both DNA and β-catenin activates Wnt-responsive genes. LEF-1 contains a single High Mobility Group (HMG) domain, which binds, bends, and distorts the minor groove of its cognate DNA. In the absence of DNA and β-catenin, the helix I and the C-terminal end of Helix III of the LEF-1 HMG domain are unstable, fluctuating on the millisecond to microsecond timescale. This region cooperatively folds upon DNA binding. This disorder appears to be a hallmark of HMG domains that bind specific DNA sequences, as opposed to proteins containing multiple HMG domains that recognize DNA structure rather than DNA sequence [271]. A final type of cell signaling regulated transcription factor includes Smad proteins, which are regulated by TGFβ/BMP signaling. These transcription factors are composed of two structured domains separate by an intrinsically disordered linker, allowing the protein structure to range between compact and extended structures [325]. Smad dynamics are hypothesized to be important for modulating Smad function and thus signal transduction.

Most gene-specific transcription factors regulate transcription by recruiting components of general transcription activation or repression complexes. These components also include IDPs/IDRs. To continue the example of LEF-1, in the absence of Wnt signaling LEF-1 binds the corepressor TLE (termed Groucho in *Drosophila*). Groucho is composed of structured domains near both termini, and a central disordered domain that prevents promiscuous binding and unrestrained repression of transcription [272].

*Termination/intracellular trafficking* Many cell signaling pathways rely on vesicle trafficking to terminate cell signaling and/or recycle the receptor proteins [326]. In neurotransmission, signaling molecules are also released from the upstream neuron by vesicles fusing with the axon terminus. IDPs/IDRs participate in vesicle release and recycling at nerve terminals (reviewed in Snead 2019). Long disordered regions mediate protein–protein interactions and are often located adjacent to catalytic domains [327, 328]. As discussed above, many disordered regions also act as lipid curvature sensors, which is detected by the intrinsically disordered amphipathic region of the GTPase-activating protein ArfGAP1. This region acts as an amphipathic lipid-packing sensor, forming α-helices upon binding highly curved membranes [327].

## Conclusions

Intrinsically disordered proteins play many diverse, yet critical roles in cell signaling pathways. Signaling imposes many logistical demands on a cell, requiring mechanisms to amply, integrate, differentiate, and propagate signals, as well as to generate unique responses to similar signals with overlapping gene expression patterns. IDPs/IDRs are uniquely suited to solving these problems, as demonstrated by several examples detailed in this review (Table 1). The many advantages conferred by disorder to cell signaling cascades means that (1) understanding signaling required definition of the roles disorder plays in each pathway, (2) many more examples of disordered proteins in cell signaling pathways are likely to be discovered, and (3) more mechanisms by which disorder functions remain to be elucidated.

**Table 1:** Examples of regulatory mechanisms, enabled by intrinsic disorder, that contribute to cell signaling

Cell signaling requirement	ID-enabled mechanism	Example in this review
Signal diversification/specificity generation	Multi-protein binding	Reck-Fz-Wnt
	Varying IDRs via gene duplication	Wnt-Fz
		Connexins
	Post-translational modifications and/or alternative splicing	CXCR4
		GPCR-G protein
		VEGF versus VEGFB isoforms
Signal passage through a membrane	Binding-induced folding	EGFR
Integration of multiple inputs to diversify responses	Binding-induced folding	Glucocorticoid receptor EGFR
	Allostery	EGFR
	Post-translational modification	PTEN
Signal amplification	Phase separation	EGFR
	Scaffold-mediated concentration of components	Axin
		Gab2
Signal propagation	Post-translational modification	EGFR
	Spatial control of protein binding/orientation	Ras EGFR
Graded or differential responses from the same protein	Spatial control of protein binding	NMDA receptor
	Splicing and post-translational modifications	Glucocorticoid receptor

The importance of disorder is highlighted by its presence in cell signaling proteins from all kingdoms of life (animals, plants, bacteria, fungi), in every category of cell signaling pathways (autocrine, juxtacrine, intracrine, paracrine, and endocrine) and at each stage (ligand, receptor, transducer, effector, terminator) in the cell signaling process. Clearly, any particular cell signaling pathway cannot be fully described without understanding the mechanisms by which intrinsically disordered protein regions contribute to that pathway. Understanding these mechanisms requires not only understanding the statistics of the conformational ensembles generated by intrinsically disordered protein regions [320], but also defining how alternative splicing, PTMs, mutation, ligand binding, effector protein binding, and changes in sub-cellular location can dynamically alter these ensembles.

## Data Availability

All data analyzed as part of this study are included in this article.

## Abbreviations

ACE2: Angiotensin-converting enzyme 2
APC: Adenomatous polyposis coli
AS: Alternative splicing
CK1: Casein kinase 1
CP12: Chloroplast protein 12
CTD: Cytoplasmic C-terminal intrinsically disordered domain
DBR: Disordered binding region
Dsh/Dvl: Disheveled
ELM: EGFR: Epidermal growth factor receptor; eukaryotic linear motif
ER: Estrogen receptor
ERE: Estrogen receptor element
ERD14: Early response to dehydration 14
ERK: Extracellular signal-regulated kinase
Fz: Frizzled
Gab2: Grb2-associated binder 2
GAP: GTP-ase accelerating proteins
GPCR: G protein coupled receptor
GSK3: Glycogen synthase kinase 3
GR: Glucocorticoid receptor
GRB2: Growth factor receptor-bound protein 2
HSPGs: Heparan sulfate proteoglycans
IDP: Intrinsically disordered protein
IDR: Intrinsically disordered region
LM: Linear motif
LMD: Large multi-site docking protein
LRP5/6: Lipo-related receptor protein 5/6
mBDNF: Mature BDNF
MoRF: Moleular recognition feature
NMDA: N-methyl-D-aspartate
PI3K: Phosphatidylinositol 3-kinase
NR3: Nuclear receptor subfamily 3
PKC: Protein kinase C
PTEN: Phosphate and Tensin homologue
PTM: Post-translational modification
SH2: Sarc homology 2
SLiM: Short linear motif
TCF/LEF: T-cell factor/lymphoid enhancer-binding factor
TGFβ/BMP: Transforming growth factor beta/bone morphogenic protein
TLE: Transducin-like enhancer of split
UVR8: UV-B resistance 8
VEGF: Vascular endothelial growth factor

## References

[1] Brown CJ, Takayam S, Campen AM, Vise P, Marshall TW, Oldfield CJ (2002). Evolutionary rate heterogeneity in proteins with long disordered regions. J Mol Evol.

[2] Cortese MS, Uversky VN, Dunker AK (2008). Intrinsic disorder in scaffold proteins: getting more from less. Prog Biophys Mol Biol.

[3] Dunker AK, Lawson JD, Brown CJ, Williams RM, Romero P, Oh JS (2001). Intrinsically disordered protein. J Mol Graph Model.

[4] Dunker AK, Garner E, Guilliot S, Romero P, Albrecht K, Hart J (1998). Protein disorder and the evolution of molecular recognition: theory, predictions and observations. Pac Symp Biocomput.

[5] Dunker AK, Obradovic Z, Romero P, Kissinger C, Villafranca JE (1997). On the importance of being disordered. Protein Data Bank Q Newsl.

[6] Romero P, Obradovic Z, Li X, Garner EC, Brown CJ, Dunker AK (2001). Sequence complexity of disordered protein. Proteins.

[7] Wright PE, Dyson HJ (1999). Intrinsically unstructured proteins: re-assessing the protein structure-function paradigm. J Mol Biol.

[8] Dunker AK, Obradović Z (2001). The protein trinity - linking function and disorder. Nat Biotechnol.

[9] Dunker AK, Brown CJ, Lawson JD, Iakoucheva LM, Obradović Z (2002). Intrinsic disorder and protein function. Biochemistry.

[10] Dunker AK, Cortese MS, Romero P, Iakoucheva LM, Uversky VN (2005). Flexible nets: the roles of intrinsic disorder in protein interaction networks. FEBS J.

[11] Daughdrill GW, Pielak GJ, Uversky VN, Cortese MS, Dunker AK, Buchner J, Kiefhaber T (2005). Natively disordered proteins. Handbook of protein folding.

[12] Dyson HJ, Wright PE (2002). Coupling of folding and binding for unstructured proteins. Curr Opin Struct Biol.

[13] Wright DHJPE (2005). Intrinsically unstructured proteins and their functions. Nat Rev Mol Cell Biol.

[14] Iakoucheva LM, Brown CJ, Lawson JD, Obradović Z, Dunker AK (2002). Intrinsic disorder in cell-signaling and cancer-associated proteins. J Mol Biol.

[15] Plaxco KW, Gross M (1997). Cell biology. The importance of being unfolded. Nature.

[16] Pontius BW (1993). Close encounters: why unstructured, polymeric domains can increase rates of specific macromolecular association. Trends Biochem Sci.

[17] Uversky VN, Oldfield CJ, Dunker AK (2005). Showing your ID: intrinsic disorder as an ID for recognition, regulation and cell signaling. J Mol Recognit.

[18] Schulz GE, Balaban M (1979). Nucleotide binding proteins. Molecular mechanism of biological recognition.

[19] Borgia A, Borgia MB, Bugge K, Kissling VM, Heidarsson PO, Fernandes CB (2018). Extreme disorder in an ultrahigh-affinity protein complex. Nature.

[20] Borg M, Mittag T, Pawson T, Tyers M, Forman-Kay JD, Chan HS (2007). Polyelectrostatic interactions of disordered ligands suggest a physical basis for ultrasensitivity. Proc Natl Acad Sci USA.

[21] Permyakov SE, Millett IS, Doniach S, Perkyakov EA, Uversky VN (2003). Natively unfolded C-terminal domain of caldesmon remains substantially unstructured after the effective binding to calmodulin. Proteins.

[22] Sigalov AB, Zhuravleva A, Orekhov VY (2007). Binding of intrinsically disordered proteins is not necessarily accompanied by a structural transition to a folded form. Biochimie.

[23] Sigalov AB (2010). Protein intrinsic disorder and oligomericity in cell signaling. Mol Biosyst.

[24] Fuxreiter M, Simon I, Bondos S (2011). Dynamic protein-DNA recognition: beyond what can be seen. Trends Biochem Sci.

[25] Fuxreiter M, Tompa P (2012). Fuzzy complexes: a more stochastic view of protein function. Adv Exp Med Biol.

[26] Launay H, Receveur-Brechot V, Carriere F, Gontero B (2019). Ochestration of algal metabolism by protein disorder. Arch Biochem Biophys.

[27] Kreiger JM, Fusco G, Lewitzky M, Simister PC, Marchant J, Camilloni C (2014). Conformational recognition of an intrinsically disordered protein. Biophys J.

[28] Ma B, Nussinov R (2009). Amplification of signaling via cellular allosteric relay and protein disorder. Proc Natl Acad Sci USA.

[29] Misiura MM, Kolomeisky AB (2002). Role of intrinsically disordered regions in acceleration of protein-protein association. J Phys Chem B.

[30] Flock T, Hauser AS, Lund N, Gloriam DE, Balaji S, Babu MM (2017). Selectivity determinants of GPCR-G-protein binding. Nature.

[31] Zhu L, Anslyn EV (2006). Signal amplification by allosteric catalysis. Angew Chem Int Ed Engl.

[32] Li L, Uversky VN, Dunker AK, Meroueh SO (2007). A computational investigation of allostery in the catabolite activator protein. J Am Chem Soc.

[33] Hilser VJ, Thompson EB (2007). Intrinsic disorder as a mechanism to optimize allosteric coupling in proteins. Proc Natl Acad Sci USA.

[34] Martin-Yken H, Francois JM, Zerbib D (2016). Knr4: a disordered hub protein at the heart of fungal cell wall signalling. Cell Microbiol.

[35] Gough J, Dunker AK (2013). Sequences and topology: disorder, modularity, and post/pre-translation modifications. Curr Opin Struct Biol.

[36] Kawamura H, Li X, Harper SJ, Bates DO, Claesson-Welsh L (2008). Vascular endothelial growth factor (VEGF)-A165b is a weak in vitro agonist for VEGF receptor-2 due to lack of coreceptor binding and deficient regulation of kinase activity. Cancer Res.

[37] Li J, White JT, Saavedra H, Wrabl JO, Motlagh HN, Liu K, Sower J (2017). Genetically tunable frustration controls allostery in an intrinsically disordered transcription factor. eLife.

[38] Dunker AK, Silman I, Uversky VN, Sussman JL (2008). Function and structure of inherently disordered proteins. Curr Opin Struct Biol.

[39] Zhou J, Zhao S, Dunker AK (2018). Intrinsically disordered proteins link alternative splicing and post-translational modifications to complex cell signaling and regulation. J Mol Biol.

[40] Romero PR, Zaidi S, Fang YY, Uversky VN, Radivojac P, Oldfield CJ (2006). Alternative splicing in concert with protein intrinsic disorder enables increased functional diversity in multicellular organisms. Proc Natl Acad Sci USA.

[41] Iakoucheva LM, Radivojac P, Brown CJ, O'Connor TR, Sikes JG, Obradovic Z, Dunker AK (2004). The importance of intrinsic disorder for protein phosphorylation. Nucleic Acids Res.

[42] Gao J, Xu D. Correlation between posttranslational modification and intrinsic disorder in protein. In: Pacific symposium on Biocomputing;2012. p. 94–103. PMC5120255 22174266

[43] Cheung P, Allis CD, Sassone-Corsi P (2000). Signaling to chromatin through histone modifications. Cell.

[44] Strahl BD, Allis CD (2000). The language of covalent histone modifications. Nature.

[45] Benayoun BA, Veitia RA (2009). A post-translational modification code for transcription factors: sorting through a sea of signals. Trends Cell Biol.

[46] Calman DR, Brunet A (2008). The FoxO code. Oncogene.

[47] Gamble MJ, Freedman LP (2002). A coactivator code for transcription. Trends Biochem Sci.

[48] Lothrop AP, Torres MP, Fuchs SM (2013). Deciphering post-translational modification codes. FEBS Lett.

[49] Meek DW, Anderson CW (2009). Post-translational modiication of p53” cooperative integrators of function. Cold Spring Harb Perspect Biol.

[50] Xu Y (2003). Regulation of p53 responses by post-translational modifications. Cell Death Differ.

[51] Yang XJ (2005). Multisite protein modification and intramolecular signaling. Oncogene.

[52] Pejaver V, Hsu W-L, Xin F, Dunker AK, Uversky VN, Radivojac P (2014). The structural and functional signatures of proteins that undergo multiple events of post-translational modification. Protein Sci.

[53] Morrison EA, Bowerman S, Sylvers KL, Wereszczynski J, Musselman CA (2018). The conformation of the histone H3 tail inhibits association of the BPTG PHD finger with the nucleosome. eLife.

[54] Uversky VN, Giuliani A (2021). Networks of networks: an essay on multi-level biological organization. Front Genet.

[55] Bai Y (2003). Hidden intermediates and Levinthal paradox in the folding of small proteins. Biochem Biophys Res Commun.

[56] Baldwin RL (1995). The nature of protein folding pathways: the classical versus the new view. J Biomol NMR.

[57] Baldwin RL, Rose GD (1999). Is protein folding hierarchic? II. Folding intermediates and transition states. Trends Biochem Sci.

[58] Christensen H, Pain RH (1991). Molten globule intermediates and protein folding. Eur Biophys J.

[59] Fink AL (1995). Compact intermediate states in protein folding. Annu Rev Biophys Biomol Struct.

[60] Kim PS, Baldwin RL (1990). Intermediates in the folding reactions of small proteins. Annu Rev Biochem.

[61] Matthews CR (1993). Pathways of protein folding. Annu Rev Biochem.

[62] Ptitsyn OB (1995). Molten globule and protein folding. Adv Protein Chem.

[63] Fink AL, Oberg KA (1998). Seshadri S, Discrete intermediates versus molten globule models for protein folding: characterization of partially folded intermediates of apomyoglobin. Fold Des.

[64] Dolgikh DA, Kolomiets AP, Ptitsyn OB (1984). 'Molten-globule' state accumulates in carbonic anhydrase folding. FEBS Lett.

[65] Dobson CM (1992). Unfolded proteins, compact states and molten globules. Curr Opin Struct Biol.

[66] Ohgushi M, Wada A (1983). 'Molten-globule state': a compact form of globular proteins with mobile side-chains. FEBS Lett.

[67] Uversky VN, Ptitsyn OB (1994). "Partly folded" state, a new equilibrium state of protein molecules: four-state guanidinium chloride-induced unfolding of beta-lactamase at low temperature. Biochemistry.

[68] Uversky VN, Ptitsyn OB (1996). Further evidence on the equilibrium "pre-molten globule state": four-state guanidinium chloride-induced unfolding of carbonic anhydrase B at low temperature. J Mol Biol.

[69] Uversky VN (2003). Protein folding revisited. A polypeptide chain at the folding-misfolding-nonfolding cross-roads: which way to go?. Cell Mol Life Sci.

[70] Uversky VN (2013). Unusual biophysics of intrinsically disordered proteins. Biochim Biophys Acta.

[71] Uversky VN (2016). Paradoxes and wonders of intrinsic disorder: complexity of simplicity. Intrinsically Disord Proteins.

[72] DeForte S, Uversky VN (2016). Order, disorder, and everything in between. Molecules.

[73] Uversky VN (2013). A decade and a half of protein intrinsic disorder: biology still waits for physics. Protein Sci.

[74] Uversky VN (2013). Intrinsic disorder-based protein interactions and their modulators. Curr Pharm Des.

[75] Jakob U, Kriwacki R, Uversky VN (2014). Conditionally and transiently disordered proteins: awakening cryptic disorder to regulate protein function. Chem Rev.

[76] Campen A, Williams RM, Brown CJ, Meng J, Uversky VN, Dunker AK (2008). TOP-IDP-scale: a new amino acid scale measuring propensity for intrinsic disorder. Protein Pept Lett.

[77] Garner E, Cannon P, Romero P, Obradovic Z, Dunker AK (1998). Predicting disordered regions from amino acid sequence: common themes despite differing structural characterization. Genome Inform Ser Workshop Genome Inform.

[78] Radivojac P, Iakoucheva LM, Oldfield CJ, Obradovic Z, Uversky VN, Dunker AK (2007). Intrinsic disorder and functional proteomics. Biophys J.

[79] Uversky VN, Dunker AK (1804). Understanding protein non-folding. Biochim Biophys Acta.

[80] Vacic V, Uversky VN, Dunker AK, Lonardi S (2007). Composition Profiler: a tool for discovery and visualization of amino acid composition differences. BMC Bioinformatics.

[81] Williams RM, Obradovic Z, Mathura V, Braun W, Garner EC, Young J, et al. The protein non-folding problem: amino acid determinants of intrinsic order and disorder. In: Pacific symposium on biocomputing; 2001. p. 89–100. 10.1142/9789814447362_0010 11262981

[82] Xie Q, Arnold GE, Romero P, Obradovic Z, Garner E, Dunker AK (1998). The sequence attributes method for determining relationships between sequence and protein disorder. Genome Inform.

[83] Darling AL, Uversky VN (2017). Intrinsic disorder in proteins with pathogenic repeat expansions. Molecules.

[84] Dosztányi Z, Chen J, Dunker AK, Simon I, Tompa P (2006). Disorder and sequence repeats in hub protein and their implications for network evolution. J Proteome Res.

[85] Jorda J, Xue B, Uversky VN, Kajava AV (2010). Protein tandem repeats—the more perfect, the less structured. FEBS J.

[86] Tompa P (2003). Intrinsically unstructured proteins evolve by repeat expansion. BioEssays.

[87] Hanson J, Paliwal KK, Litfin T, Zhou Y (2019). SPOT-Disorder2: improved protein intrinsic disorder prediction by ensembled deep learning. Genomics Proteomics Bioinform.

[88] Dosztányi Z, Csizmok V, Tompa P, Simon I (2005). IUPred: web server for the prediction of intrinsically unstructured regions of proteins based on estimated energy content. Bioinformatics.

[89] Jones DT, Cozzetto D (2015). DISOPRED3: precise disordered region predictions with annotated protein-binding activity. Bioinformatics.

[90] Peng K, Radivojac P, Vucetic S, Dunker AK, Obradovic Z (2006). Length-dependent prediction of protein intrinsic disorder. BMC Bioinform.

[91] Romero P, Obradovic Z, Kissinger C, Villagranca JE, Dunker AK. Identifying disordered regions in proteins from amino acid sequence. In: IEEE international conference on neural networks, ICNN, Houston, TX. 1997.

[92] Necci M, Piovesan D, CAID Predictors, DisProt Curators, Tosatto SCE. Critical assessment of protein intrinsic disorder prediction. BioRxiv. 2020. 10.1101/2020.08.11.245852.

[93] He B, Wang K, Liu Y, Xue B, Uversky VN, Dunker AK (2009). Predicting intrinsic disorder in proteins: an overview. Cell Res.

[94] Fan X, Kurgan L (2014). Accurate prediction of disorder in protein chains with a comprehensive and empirically designed consensus. J Biomol Struct Dyn.

[95] Hu G, Wu Z, Oldfield CJ, Wang C, Kurgan L (2019). Quality assessment for the putative intrinsic disorder in proteins. Bioinformatics.

[96] Katuwawala A, Oldfield CJ, Kurgan L (2020). Accuracy of protein-level disorder predictions. Brief Bioinform.

[97] Lieutaud P, Ferron F, Uversky AV, Kurgan L, Uversky VN, Longhi S (2016). How disordered is my protein and what is its disorder for? A guide through the "dark side" of the protein universe. Intrinsically Disord Proteins.

[98] Lieutaud P, Ferron F, Longhi S (2016). Predicting conformational disorder. Methods Mol Biol.

[99] Li J, Feng Y, Wang X, Li J, Liu W, Rong L (2015). An overview of predictors for intrinsically disordered proteins over 2010–2014. Int J Mol Sci.

[100] Liu Y, Wang X, Liu B (2019). A comprehensive review and comparison of existing computational methods for intrinsically disordered protein and region prediction. Brief Bioinform.

[101] Meng F, Uversky VN, Kurgan L (2017). Comprehensive review of methods for prediction of intrinsic disorder and its molecular functions. Cell Mol Life Sci.

[102] Peng ZL, Kurgan L (2012). Comprehensive comparative assessment of in-silico predictors of disordered regions. Curr Protein Pept Sci.

[103] Dunker AK, Obradović Z, Romero P, Garner EC, Brown CJ (2000). Intrinsic protein disorder in complete genomes. Genome Inform Ser Workshop Genome Inform.

[104] Oates ME, Romero P, Ishida T, Ghalwash M, Mizianty MJ, Xue B (2013). D(2)P(2): database of disordered protein predictions. Nucleic Acids Res.

[105] Peng Z, Yan J, Fan X, Mizianty M, Xue B, Wang K (2015). Exceptionally abundant exceptions: comprehensive characterization of intrinsic disorder in all domains of life. Cell Mol Life Sci.

[106] Uversky VN (2010). The mysterious unfoldome: structureless, underappreciated, yet vital part of any given proteome. J Biomed Biotechnol.

[107] Xue B, Dunker AK, Uversky VN (2012). Orderly order in protein intrinsic disorder distribution: disorder in 3500 proteomes from viruses and the three domains of life. J Biomol Struct Dyn.

[108] Oldfield CJ, Uversky VN, Dunker AK, Kurgan L, Salvi N (2019). Introduction to intrinsically disordered proteins and regions. Intrinsically disordered proteins: dynamics, binding, and function.

[109] Xie H, Vucetic S, Iakoucheva LM, Oldfield CJ, Dunker AK, Uversky VN (2007). Functional anthology of intrinsic disorder. 1. Biological processes and function of proteins with long disordered regions. J Proteome Res.

[110] Xie H, Vucetic S, Iakoucheva LM, Oldfield CJ, Dunker AK, Obradovic Z (2007). Functional anthology of intrinsic disorder. 3. Ligands, post-translational modifications, and diseases associated with intrinsically disordered proteins. J Proteome Res.

[111] Vucetic S, Xie H, Iakoucheva LM, Oldfield CJ, Dunker AK, Obradovic Z, Uversky VN (2007). Functional anthology of intrinsic disorder. 2. Cellular compartments, domains, technical terms, developmental processes, and coding sequence diversities correlated with long disordered regions. J Proteome Res.

[112] Alterovitz W-L, Faraggi E, Oldfield CJ, Meng J, Xue B, Huang F (2020). Many-to-one binding by intrinsically disordered protein regions. Pac Symp Biocomput.

[113] Hsu W-L, Oldfield C, Meng J, Huang F, Xue B, Uversky VN, et al. Intrinsic protein disorder and protein-protein interactions. In: Pacific symposium on biocomputing; 2012. p. 116–127. 22174268

[114] Hsu W-L, Oldfield CJ, Xue B, Meng J, Huang F, Romero P, Uversky VN, Dunker AK (2013). Exploring the binding diversity of intrinsically disordered proteins in one-to-many binding. Protein Sci.

[115] Karush F (1950). Heterogeneity of the binding sites of bovine serum albumin. J Am Chem Soc.

[116] Kriwacki RW, Hengst L, Tennant L, Reed SI, Wright PE (1996). Structural studies of p21Waf1/Cip1/Sdi1 in the free and Cdk2-bound state: conformational disorder mediates binding diversity. Proc Natl Acad Sci USA.

[117] Oldfield CJ, Meng J, Yang JY, Yang MQ, Uversky VN, Dunker AK (2008). Flexible nets: disorder and induced fit in the associations of p53 and 14-3-3- with their partners. BMC Genomics.

[118] DeForte S, Uversky VN (2017). Not an exception to the rule: the functional significance of intrinsically disordered protein regions in enzymes. Mol Biosyst.

[119] Cheng Y, Oldfield CJ, Meng J, Romero P, Uversky VN, Dunker AK (2007). Mining alpha-helix-forming molecular recognition features with cross species sequence alignments. Biochemistry.

[120] Mohan A, Oldfield CJ, Radivojac P, Vacic V, Cortese MS, Dunker AK (2006). Analysis of molecular recognition features (MoRFs). J Mol Biol.

[121] Oldfield CJ, Cheng Y, Cortese MS, Romero P, Uversky VN, Dunker KE (2005). Coupled folding and binding with alpha-helix-forming molecular recognition elements. Biochemistry.

[122] Vacic V, Oldfield CJ, Mohan A, Radivojac P, Cortese MS, Uversky VN (2007). Characterization of molecular recognition features, MoRFs, and their binding partners. J Proteome Res.

[123] Disfani FM, Hsu WL, Mizianty MJ, Oldfield CJ, Xue B, Dunker AK (2012). MoRFpred, a computational tool for sequence-based prediction and characterization of short disorder-to-order transitioning binding regions in proteins. Bioinformatics.

[124] Dosztányi Z, Meszaros B, Simon I (2009). ANCHOR: web server for predicting protein binding regions in disordered proteins. Bioinformatics.

[125] Mészáros B, Simon I, Dosztanyi Z (2009). Prediction of protein binding regions in disordered proteins. PLoS Comput Biol.

[126] Fang C, Noguchi T, Tominaga D, Yamana H (2013). MFSPSSMpred: identifying short disorder-to-order binding regions in disordered proteins based on contextual local evolutionary conservation. BMC Bioinform.

[127] Malhis N, Gsponer J (2015). Computational identification of MoRFs in protein sequences. Bioinformatics.

[128] Malhis N, Wong ETC, Nassar R, Gsponer J (2015). Computational identification of MoRFs in protein sequences using hierarchical application of Bayes rule. PLoS ONE.

[129] Malhis N, Jacobson M, Gsponer J (2016). MoRFchibi SYSTEM: software tools for the identification of MoRFs in protein sequences. Nucleic Acids Res.

[130] Xue B, Dunker AK, Uversky VN (2010). Retro-MoRFs: identifying protein binding sites by normal and reverse alignment and intrinsic disorder prediction. Int J Mol Sci.

[131] Sharma R, Kumar S, Tsunoda T, Patil A, Sharma A (2016). Predicting MoRFs in protein sequences using HMM profiles. BMC Bioinform.

[132] Sharma R, Bayarjargal M, Tsunoda T, Patil A, Sharma A (2018). MoRFPred-plus: computational identification of MoRFs in protein sequences using physicochemical properties and HMM profiles. J Theor Biol.

[133] Sharma R, Raicar G, Tsunoda T, Patil A, Sharma A (2018). OPAL: prediction of MoRF regions in intrinsically disordered protein sequences. Bioinformatics.

[134] Sharma R, Sharma A, Raicar G, Tsunoda T, Patil A (2019). OPAL+: Length-specific MoRF prediction in intrinsically disordered protein sequences. Proteomics.

[135] Fang C, Mariwaki Y, Tian A, Li C, Shimizu K (2019). Identifying short disorder-to-order binding regions in disordered proteins with a deep convolutional neural network method. J Bioinform Comput Biol.

[136] Hanson J, Litfin T, Paliwal K, Zhou Y (2020). Identifying molecular recognition features in intrinsically disordered regions of proteins by transfer learning. Bioinformatics.

[137] He H (2019). Zhao J, Sun G, Computational prediction of MoRFs based on protein sequences and minimax probability machine. BMC Bioinform.

[138] Fang C, Mariwaki Y, Li C, Shimizu K (2019). MoRFPred_en: sequence-based prediction of MoRFs using an ensemble learning strategy. J Bioinform Comput Biol.

[139] Gould CM, Diella F, Via A, Puntervoll P, Gemund C, Chabanis-Davidson S (2010). ELM: the status of the 2010 eukaryotic linear motif resource. Nucleic Acids Res.

[140] Puntervoll P, Linding R, Gemund C, Chabanis-Davidson S, Mattingsdal M, Cameron S (2003). ELM server: a new resource for investigating short functional sites in modular eukaryotic proteins. Nucleic Acids Res.

[141] Fuxreiter M, Tompa P, Simon I (2007). Local structural disorder imparts plasticity on linear motifs. Bioinformatics.

[142] Davey NE, Shields DC, Edwards RJ (2006). SLiMDisc: short, linear motif discovery, correcting for common evolutionary descent. Nucleic Acids Res.

[143] Edwards RJ, Davey NE, Shields DC (2007). SLiMFinder: a probabilistic method for identifying over-represented, convergently evolved, short linear motifs in proteins. PLoS ONE.

[144] Mooney C, Pollastri G, Shields DC, Haslam NJ (2012). Prediction of short linear protein binding regions. J Mol Biol.

[145] Fields S, Song O (1989). A novel genetic system to detect protein-protein interactions. Nature.

[146] Barabási AL, Oltvai ZN (2004). Network biology: understanding the cell's functional organization. Nat Rev Genet.

[147] Jeong H, Mason SP, Barabási AL, Oltvai ZN (2001). Lethality and centrality in protein networks. Nature.

[148] Barabási AL, Bonabeau E (2003). Scale-free networks. Sci Am.

[149] Hasty J, Collins JJ (2001). Protein interactions. Unspinning the web. Nature.

[150] Oldfield CJ, Peng Z, Kurgan L (2020). Disordered RNA-binding region prediction with DisoRDPbind. Methods Mol Biol.

[151] Peng Z, Kurgan L (2015). High-throughput prediction of RNA, DNA and protein binding regions mediated by intrinsic disorder. Nucleic Acids Res..

[152] Peng Z, Wang C, Uversky VN, Kurgan L (2017). Prediction of disordered RNA, DNA, and protein binding regions using DisoRDPbind. Methods Mol Biol.

[153] Katuwawala A, Peng Z, Yang J, Kurgan L (2019). Computational prediction of MoRFs, short disorder-to-order transitioning protein binding regions. Comput Struct Biotechnol J.

[154] Uversky VN (2020). New technologies to analyze protein function: an intrinsic disorder perspective. F1000 Research.

[155] Receveur-Brechot V, Bourhis JM, Uversky VN, Canard B, Longhi S (2006). Assessing protein disorder and induced folding. Proteins.

[156] Sormanni P, Piovesan D, Heller GT, Bonomi M, Kukic P, Camilloni C (2017). Simultaneous quantification of protein order and disorder. Nat Chem Biol.

[157] Schramm A, Bignon C, Brocca S, Grandori R, Santambrogio C, Longhi S (2019). An arsenal of methods for the experimental characterization of intrinsically disordered proteins: how to choose and combine them?. Arch Biochem Biophys.

[158] Uversky VN (2015). Biophysical methods to investigate intrinsically disordered proteins: avoiding an "elephant and blind men" situation. Adv Exp Med Biol.

[159] Uversky VN, Dunker AK (2012). Multiparametric analysis of intrinsically disordered proteins: looking at intrinsic disorder through compound eyes. Anal Chem.

[160] Grybowska EA (2018). Calcium-binding proteins with disordered structure and their role in secretion, storage, and cellular signaling. Biomolecules.

[161] Buchner S, Schlundt A, Lassak J, Sattler M, Jung K (2015). Structural and functional analysis of the signal-transducing linker in the pH-responsive one-component system CadC of *Escherichia coli*. J Mol Biol.

[162] Camacho IS, Theisen A, Johannissen LO, Diaz-Raos LA, Christie JM, Jenkins GI (2019). Native mass spectrometry reveals the conformational diversity of the UVR8 photoreceptor. Proc Natl Acad Sci USA.

[163] Emenecker RJ, Holehouse AS, Strader LC (2020). Emerging roles for phase separation in plants. Dev Cell.

[164] Zamora-Briseno JA, Pereira-Santana A, Reyes-Hernandez SJ, Cerqueda-Garcia D, Castano E, Rodriguez-Zapata LC (2021). Towards an understanding of the role of intrinsic protein disorder on plant adaptation to environmental challenges. Cell Stress Chaperones.

[165] Alvarado A, Berens W, Josenhans C (2019). Protein activity sensing in bacteria regulating metabolism and motility. Front Microbiol.

[166] Briard B, Place DE, Kanneganti TD (2020). DNA sensing in the innate immune response. Physiology.

[167] Campbell KL, Haspel N, Gath C, Kurniatash N, Akkiraju I, Stuffers N (2021). Protein hormone fragmentation in intercellular signaling: hormones as nested information systems. Biol Reprod.

[168] Lüscher C, Malenka RC (2012). NMDA receptor-dependent long-term potentiation and long-term depression (LTP/LTD). Cold Spring Harb Perspect Biol.

[169] Warnet XL, Krog HB, Sevillano-Quispe OG, Poulsen H, Kjaergaard M (2020). The C-terminal domains of the NMDA receptor: how intrinsically disordered tails affect signaling, plasticity, and disease. Eur J Neurosci.

[170] De Geus TJ, Patijn J, Joosten EAJ (2020). Qualitative review on N-methyl-D-aspartate receptor expression in rate spinal cord during the postnatal development: implications for central sensitization and pain. Dev Neurobiol.

[171] Lussier MP, Sanz-Clemente A, Roche KW (2015). Dynamic regulation of N-methyl-d-aspartate (NMDA) and a-amino-3-hydroxy-5-methyl-4-isoeazoleproprionic acid (AMPA) receptors by posttranslational modifications. J Biol Chem.

[172] Niklas KJ, Bondos SE, Dunker AK, Newman SA (2015). Rethinking gene regulatory networks in light of alternative splicing, intrinsically disordered protein domains, and post-translational modifications. Front Cell Dev Biol.

[173] Creamer TP (2020). Calcineurin. Cell Commun Signal.

[174] Moosa MM, Ferreon JC, Ferreon ACM (2020). Ligand interactions and the protein order-disorder energetic continuum. Sem Cell Dev Biol.

[175] Toto A, Malagrino F, Visconti L, Troilo F, Pagano L, Brunori M, Jemth P, Gianni S (2020). Templated folding of intrinsically disordered proteins. J Biol Chem.

[176] Motlagh HN, Li J, Thompson EB, Hilser VJ (2012). Interplay between allostery and intrinsic disorder in an ensemble. Biochem Soc Trans.

[177] Li J, Motlaugh HN, Chakuroff C, Thompson EB, Hilser VJ (2012). Thermodynamic dissection of the intrinsically disordered N-terminal domain of human glucocorticoid receptor. J Biol Chem.

[178] Kumar R, Thompson EB (2019). Role of phosphorylation in the modulation of the Glucocorticoid Receptor’s Intrinsically disordered domain. Biomolelcules.

[179] Obradovic Z, Peng K, Vucetic S, Radivojac P, Dunker AK (2005). Exploiting heterogeneous sequence properties improves prediction of protein disorder. Protein.

[180] Obradovic Z, Peng K, Vucetic S, Radivojac P, Brown CJ, Dunker AK (2003). Predicting intrinsic disorder from amino acid sequence. Protein.

[181] Xue B, Dunbrack RL, Williams RW, Dunker AK, Uversky VN (2010). PONDR-FIT: a meta-predictor of intrinsically disordered amino acids. Biochim Biophys Acta.

[182] Mészáros B, Erdos G, Dosztányi Z (2018). IUPred2A: Context-dependent prediction of protein disorder as a function of redox state and protein binding. Nucl Acids Res.

[183] Khan SH, McLaughlin WA, Kumar R (2017). Site-specific phosphorylation regulates the structure and function of an intrinsically disordered domain of the glucocorticoid receptor. Sci Rep.

[184] Duma D, Cidlowski JA (2010). Generating diversity in glucocorticoid receptor signaling: mechanisms, receptor isoforms, and post-translational modifications. Horm Mol Biol Clin Investig.

[185] Leventhal SM, Lim D, Green TL, Cantrell AE, Cho K, Greenhalgh DG (2019). Uncovering a multitude of human glucocorticoid receptor variants: an expansive survey of a single gene. BMC Genet.

[186] Vandevyver S, Dejager L, Libert C (2014). Comprehensive overview of the structure and regulation of the glucocorticoid receptor. Endocr Rev.

[187] Mingo J, Luna S, Gaafar A, Nunes-Xavier E, Torices L, Mosteiro L (2019). Precise definition of PTEN C-terminal epitopes and its implications in clinical oncology. Precision Oncol.

[188] Lesli NR, Batty IH, Maccario H, Davidson L, Downes CP (2008). Understanding PTEN regulation: PIP2, polarity and protein stability. Oncogene.

[189] Odriozola L, Singh G, Hoang T, Chang AM (2007). Regulation of PTEN activity by its carboxyl-terminal autoinhibitory domain. J Biol Chem.

[190] Rahdar M, Inoue T, Zhang J, Vazquez F, Devreotes PN (2009). A phosphorylation-dependent intramolecular interactions regulates the membrane association and activity of the tumor suppressor PTEN. Proc Natl Acad Sci USA.

[191] Macario H, Perera NM, Davidson L, Downes CP, Leslie NR (2007). PTEN is destabilized by phosphorylation on Thr366. Biochem J.

[192] Malaney P, Uversky VN, Davé V (2015). Identification of intrinsically disordered region in PTEN and delineation of its function via a network approach. Methods.

[193] Masson GR, Williams RL (2020). Structural mechanisms of PTEN regulation. Cold Spring Harbor Perspect Med.

[194] Hopkins BD, Fine B, Steinbach N, Dendy M, Rapp Z, Shaw J (2013). A secreted PTEN phosphatase that enters cells to alter signaling and survival. Science.

[195] Masson GR, Perisic O, Burke JE, Williams RL (2016). The intrinsically disordered tails of PTEN and PTEN-L have distinct roles in regulating substrate specificity and membrane activity. Biochem J.

[196] Icer MA, Gezmen-Karadag M (2018). The multiple functions and mechanisms of osteopontin. Clin Biochem.

[197] O’Regan A, Berman JS (2000). Osteopontin: a key cytokine in cell-mediated and granulomatous inflammation. Int J Exp Pathol.

[198] Kurzbach D, Platzer G, Schwarz TC, Henen MA, Konrat R, Hinderberger D (2013). Cooperative unfolding of compact conformations of the intrinsically disordered protein osteopontin. Biochemistry.

[199] Robinson CJ, Stringer SE (2001). The splice variants of vascular endothelial growth factor (VEGF) and their reeptors. J Cell Sci.

[200] Woolard J, Wang WY, Bevan HS, Qiu Y, Morbidelli L, Pritchard-Jones RO (2004). VEGF165b, an inhibitory vascular endothelial growth factor splice variant: mechanism of action in vivo effect on aniogenesis and endogenous protein expression. Cancer Res.

[201] Boudria A, Faycal CA, Jia T, Gout S, Keramidas M, Didier C (2019). VEGF165b, a splice variant of VEGF-A, promotes lung tumor progression and escape from anti-angiogenic therapies through a b1 integrin/VEGFR autocrine loop. Oncogene.

[202] Bjarnadottir TK, Gloriam DE, Hellstrand SH, Kristiansson H, Fredriksson R, Schioth HB (2006). Comprehensive repertoire and phylogenetic analysis of the G protein-coupled receptors in human and mouse. Genomics.

[203] Anantharaman V, Abhiman S, de Souza RF, Aravind L (2011). Comparative genomics uncovers novel structural and functional features of the heterotrimeric GTPase signaling system. Gene.

[204] Fredriksson R, Lagerstrom MC, Lundin LG, Schioth HB (2003). The G-protein-coupled receptors in the human genome form five main families. Phylogenetic analysis, paralogon groups, and fingerprints. Mol Pharmacol.

[205] Southan C, Sharman JL, Benson HE, Faccenda E, Pawson AJ, Alexander SP (2016). The IUPHAR/BPS Guide to PHARMACOLOGY in 2016: towards curated quantitative interactions between 1300 protein targets and 6000 ligands. Nucl Acids Res.

[206] Engelhardt S, Rochais F (2007). G proteins: more than transducers of receptor-generated signals?. Circ Res.

[207] Gilman AG (1987). G proteins: transducers of receptor-generated signals. Annu Rev Biochem.

[208] Bourne HR, Sanders DA, McCormick F (1990). The GTPase superfamily: a conserved switch for diverse cell functions. Nature.

[209] Simon MI, Strathmann MP, Gautam N (1991). Diversity of G proteins in signal transduction. Science.

[210] Syrovatkina V, Alegre KO, Dey R, Huang XY (2016). Regulation, signaling, and physiological functions of G-proteins. J Mol Biol.

[211] Downes GB, Gautam N (1999). The G protein subunit gene families. Genomics.

[212] Temple BR, Jones CD (2010). Jones AM (2010) Evolution of a signaling nexus constrained by protein interfaces and conformational States. PLoS Comput Biol.

[213] Fonin AV, Darling AL, Kuznetsova IM, Tuoverov KK, Uversky VN (2019). Multi-functionality of proteins involved in GPCR and G protein signaling: making sense of structure-function continuum with intrinsic disorder-based proteoforms. Cell Mol Life Sci.

[214] Sprenger WW, Hoff WD, Armitage JP, Hellingwerf KJ (1993). The eubacterium Ectothiorhodospira halophila is negatively phototactic, with a wavelength dependence that fits the absorption spectrum of the photoactive yellow protein. J Bacteriol.

[215] Borgstahl GE, Williams DR, Getzoff ED (1995). 1.4 Å structure of photoactive yellow protein, a cytosolic photoreceptor: unusual fold, active site, and chromophore. Biochemistry.

[216] Hoff WD, van Stokkum IH, van Ramesdonk HJ, van Brederode ME, Brouwer AM, Fitch JC (1994). Measurement and global analysis of the absorbance changes in the photocycle of the photoactive yellow protein from Ectothiorhodospira halophila. Biophys J.

[217] Meyer TE (1985). Isolation and characterization of soluble cytochromes, ferredoxins and other chromophoric proteins from the halophilic phototrophic bacterium Ectothiorhodospira halophila. Biochim Biophys Acta.

[218] Van Beeumen JJ, Devreese BV, van Bun SM, Hoff WD, Hellingwerf KJ, Meyer TE (1993). Primary structure of a photoactive yellow protein from the phototrophic bacterium Ectothiorhodospira halophila, with evidence for the mass and the binding site of the chromophore. Protein Sci.

[219] Meyer TE, Yakali E, Cusanovich MA, Tollin G (1987). Properties of a water-soluble, yellow protein isolated from a halophilic phototrophic bacterium that has photochemical activity analogous to sensory rhodopsin. Biochemistry.

[220] Rubinstenn G, Vuister GW, Mulder FAA, Düx PE, Boelens R, Hellingwerf KJ (1998). Structural and dynamic changes of photoactive yellow protein during its photocycle in solution. Nat Struct Biol.

[221] Janmey PA, Miller RT (2011). Mechanisms of mechanical signaling in development and disease. J Cell Sci.

[222] Fedorhak GR, Kaminski A, Lammerding J (2014). Cellular mechanosensing: getting to the nucleus of it all. Prog Biophys Mol Biol.

[223] Bush M, Alhanshali BM, Qian S, Stanley CB, Heller WT, Matsui T (2019). An ensemble of flexible conformations underlies mechanotransduction by the cadherin-catenin adhesion complex. Proc Natl Acad Sci USA.

[224] Hotta K, Ranganathan S, Liu R, Wu F, Machiyama H, Gao R (2014). Biophysical properties of intrinsically disordered p130Cas substrate domain—implication in mechanosensing. PLoS Comput Biol.

[225] Goult BT, Yan J, Scwartz MA (2018). Talin as a mechanosensitive signaling hub. J Cell Biol.

[226] Margadant F, Chew LL, Hu X, Bate N, Zhang X, Sheetz M (2011). Mechanotransduction in vivo by repeated Talin stretch-relaxation events depends upon Vinculin. PLoS Biol.

[227] Hong W, Jiao W, Hu J, Zhang J, Liu C, Fu X (2005). Perplasmic protein HdeA exhibits chaperone-like activity exclusively within stomach pH range by transforming into disordered conformation. J Biol Chem.

[228] Yu ZY, Wang X, Zhang LS (2018). Structural and functional dynamics of dehydrins: a plant protector protein under abiotic stress. Int J Mol Sci.

[229] Yokoyama T, Ohkubo T, Kamiya K, Hara M (2020). Cryoprotective activity of Arabidopsis KS-type dehydrin depends on the hydrophobic amino acids of the two active segments. Arch Biochem Biophys.

[230] Murvai N, Kalmar L, Szalaine Agoston B, Szabo B, Tantos A, Csikos G (2020). Interplay of structural disorder and short binding elements in the cellular chaperone function of plant dehydrin ERD14. Cells.

[231] Aksoy H, Karadag AS, Wollina U (2020). Angiotensin II receptors: Impact for COVID-19 severity. Dermatol Ther.

[232] Dahlman-Wright K, Cavailles V, Fuqua SA, Jordan VC, Katzenellenbogen JA, Korach KS (2006). International union of pharmacology. LXIV Estrogen receptors Pharmacol Rev.

[233] Lu NZ, Wardell SE, Burnstein KL, Defranco D, Fuller PJ, Giguere V (2006). International union of pharmacology. LXV. The pharmacology and classification of the nuclear receptor superfamily: glucocorticoid, mineralocorticoid, progerterone, and androgen recpetors. Pharmacol Rev.

[234] O’Lone R, Frith MC, Karlsson EK, Hansen U (2004). Genomics targets of nuclear estrogen receptors. Mol Endocrinol.

[235] Fuentes N, Silveyra P (2019). Estrogen receptor signaling mechanisms. Adv Protein Chem Struct Biol.

[236] Klinge CM (2001). Estrogen receptor interaction with estrogen response elements. Nucleic Acids Res.

[237] Marino M, Galluzzo P, Ascenzi P (2006). Estrogen signaling multiple pathways to impact gene transcription. Curr Genomics.

[238] Vrtačnik P, Ostanek B, Mencej-Bedrač S, Marc J (2014). The many faces of estrogen signaling. Biochem Med (Zagreb).

[239] Göttlicher M, Heck S, Herrlich P (1998). Transcriptional cross-talk, the second mode of steroid hormone receptor action. J Mol Med (Berl).

[240] Langhorne J, Gillard S, Simon B, Slade S, Eichmann K (1989). Frequencies of CD4+ T cells reactive with Plasmodium chabaudi chabaudi: distinct response kinetics for cells with Th1 and Th2 characteristics during infection. Int Immunol.

[241] Lonard DM, O’Malley BW (2006). The expanding cosmos of nuclear receptor coactivators. Cell.

[242] Lonard DM, O’Malley BW (2007). Nuclear receptor coregulators: judges, juries, and executioners of cellular regulation. Mol Cell.

[243] Han SJ, Lonard DM, O’Malley BW (2009). Multi-modulation of nuclear receptor coactivators through posttranslational modifications. Trends Endocrinol Metab.

[244] Peng Y, Cao S, Kiselar J, Xiao X, Du Z, Hsieh A (2019). A metastable contact and structural disorder in the estrogen receptor transactivation domain. Structure.

[245] Rajbhandari P, Finn G, Solodin NM, Singarapu KK, Sahu SC, Markley JL (2012). Regulation of estrogen receptor α N-terminus conformation and function by peptidyl prolyl isomerase Pin1. Mol Cell Bol.

[246] Rajbhandari P, Szatkowski Ozers M, Solodin NM, Warren CL, Alarid ET (2015). Peptidylprolyl isomerase Pin1 directly enhances the DNA binding functions of Estrogen Receptor α. J Biol Chem.

[247] Harward SC, Hedrick NG, Hall CE, Parra-Bueno P, Milner TA, Pan E (2016). Autocrine BDNF-TrkB signalling within a single dendritic spine. Nature.

[248] Wang J, Anastasia A, Bains H, Giza JI, Clossey DG, Deng J (2020). Zinc induced structural changes in the intrinsically disordered BDNF Met prodomain confer synaptic elimination. Metallomics.

[249] Callahan R, Egan SE (2004). Notch signaling in mammary development and oncogenesis. J Mammary Gland Biol Neoplasia.

[250] Monticone G, Miele L (2021). Notch pathway: a journey from notching phenotypes to cancer immunotherapy. Adv Exp Med Biol.

[251] Wilson JJ, Kovall RA (2006). Crystal structure of the CSL-Notch-Mastermind complex bounds to DNA. Cell.

[252] Sherry KP, Das RK, Pappu RV, Barrick D (2017). Control of transcriptional activity by design of charge patterning in the intrinsically disordered RAM region of the Notch receptor. Proc Natl Acad Sci USA.

[253] Sorgen PL, Trease AJ, Spagnol G, Delmmar M, Nielsen MS (2018). Protein-protein interactions with Connexin 43” Regulation and function. Int J Mol Sci.

[254] Trease AJ, Li H, Spagnol G, Zheng L, Stauch KL, Sorgen PL (2019). Regulation of Connexin32 by ephrin receptors and T-cell protein-tyrosine phosphatase. J Biol Chem.

[255] Enz R (2012). Structure of metabotropic glutamate receptor C-terminal domains in contact with interacting proteins. Front Mol Neurosci.

[256] Nusse R, Brown A, Papkoff J, Scambler P, Shackleford G, McMahon A (1991). A new nomenclature for int-1 and related genes: the Wnt gene family. Cell.

[257] Nusse R, Clevers H (2017). Wnt/β-catenin signaling, disease, and emerging therapeutic modalities. Cell.

[258] Eubelen M, Bostaille N, Cabochette P, Gauquier A, Tebabi P, Dumitru AC (2018). A molecular mechanism for Wnt ligand-specific signaling. Science.

[259] Xue B, Romero PR, Noutsou M, Maurice MM, Rüdiger SG, William AM (2013). Stochastic machines as a colocalization mechanism for scaffold protein function. FEBS Lett.

[260] Kimelman D, Xu W (2006). beta-catenin destruction complex: insights and questions from a structural perspective. Oncogene.

[261] Radivojac P, Vacic V, Haynes C, Cocklin RR, Mohan A, Heyen JW (2010). Identification, analysis, and prediction of protein ubiquitination sites. Proteins.

[262] Prakash S, Tian L, Ratliff KS, Lehotzky RE, Matouschek A (2004). An unstructured initiation site is required for efficient proteasome-mediated degradation. Nat Struct Mol Biol.

[263] Noutsou M, Duarte AM, Anvarian Z, Didenko T, Minde DP, Kuper I (2011). Critical scaffolding regions of the tumor suppressor Axin1 are natively unfolded. J Mol Biol.

[264] Harnoš J, Cañizal MCA, Jurásek M, Kumar J, Holler C, Schambony A (2019). Dishevelled-3 conformation dynamics analyzed by FRET-based biosensors reveals a key role of casein kinase 1. Nat Commun.

[265] Hanáková K, Bernatík O, Kravec M, Micka M, Kumar J, Harnoš J (2019). Comparative phosphorylation map of Dishevelled 3 links phosphor-signatures to biological outputs. Cell Commun Signal.

[266] Burgi J, Xue B, Uversky VN, van der Groot G (2016). Intrinsic disorder in transmembrane proteins: roles in signaling and topology prediction. PLoS ONE.

[267] Dunker AK, Bondos SE, Huang F, Oldfield CJ (2015). Intrinsically disordered proteins and multicellular organisms. Semin Cell Dev Biol.

[268] Alowolodu O, Johnsnon G, Alashwal L, Addou I, Zhdanova I, Uversky VN (2016). Intrinsic disorder in spondins and some of their interacting partners. Intrinsically Disord Proteins.

[269] Xue B, Dunker AK, Uversky VN (2012). The roles of intrinsic disorder in orchestrating the Wnt-Pathway. J Biomol Struct Dyn.

[270] Zhao B, Xue B (2016). Self-regulation of functional pathways by motifs inside the disordered tails of beta-catenin. BMC Genomics.

[271] Love JL, Li X, Chung J, Dyson HJ, Wright PE (2004). The LEF high-mobility group domain undergoes a disorder-to-order transition upon formation of a complex with cognate DNA. Biochemistry.

[272] Turki-Judeh W, Courey AJ (2012). The unconserved Grouch central region is essential for viability and modulates target gene specificity. PLoS ONE.

[273] Brodsky S, Jana T, Mittelman K, Chapal M, Kumar DK, Carmi M (2020). Intrinsically disordered regions direct transcription factor in vivo binding specificity. Mol Cell.

[274] Clark S, Myers J, King A, Fiala R, Novacek J, Pearce G (2018). Multivalency regulates activity in an intrinsically disordered transcription factor. eLife.

[275] Liu J, Perumal NB, Oldfield CJ, Su EW, Uversky VN, Dunker AK (2006). Intrinsic disorder in transcription factors. Biochemistry.

[276] Tarczewska A, Greb-Markiewicz B (2019). The significance of the intrinsically disordered regions for the functions of the bHLH transcription factors. Int J Mol Sci.

[277] Vuzman D, Levy Y (2012). Intrisically disordered regions as affinity tuners in protein-DNA interactions. Mol BioSyst.

[278] Xu X, Dai Y (2010). Heparin: an intervenor in cell communication. J Cell Mol Med.

[279] Peysselon F, Richard-Blum S (2014). Heparin-protein interactions: from affinity and kinetics to biological roles. Application to an interaction network regulating angiogenesis. Matrix Biol.

[280] Koganti R, Suryawanshi R, Shukla D (2020). Heparanase, cell signaling, and viral infections. Cell Mol Life Sci.

[281] Cornish J, Chamerlain SG, Own D, Mott HR (2020). Intrinsically disordered proteins and membranes: a marriage of convenience for cell signaling?. Biochem Soc Trans.

[282] Keppel TR, Sarpong K, Murray EM, Monsey J, Zhu J, Bose R (2017). Biophysical evidence for intrinsic disorder in the C-terminal tails of the Epidermal Growth Factor Receptor (EGFR) and HER3 Receptor Tyrosine Kinases. J Biol Chem.

[283] Sigalov AB, Uversky VN (2011). Differential occurrence of protein intrinsic disorder in the cytoplasmic signaling domains of cell receptors. Self Nonself.

[284] Arkhipov A, Shan Y, Das R, Endres NF, Eastwood MP, Wemmer DE (2013). Architecture and membrane interactions of the EGF receptor. Cell.

[285] Endres NF, Das R, Smith AW, Arkhipov A, Kovacs E, Huang Y (2013). Conformation coupling across the plasma membrane in activation of the EGF receptor. Cell.

[286] Maeda R, Sato T, Okamoto K, Yanagawa M, Sako Y (2018). Lipid-protein interplay in dimerization of juxtamembrane domains of epidermal growth factor receptor. Biophys J.

[287] Eck MJ, Hahn EC (2012). EGR in limbo. Cell.

[288] Deryusheva E, Nemashkalova E, Galloux M, Richard C-A, Eléouët J-F, Kovacs D (2019). Does intrinsic disorder in proteins favor their interaction with lipids?. Proteomics.

[289] Fuglebakk Reuter N (2018). A model for hydrophobic protrusions on peripheral membrane proteins. PLoS Comput Biol.

[290] Neale C, Garia AE (2020). The plasma membrane as a competitive inhibitor and positive allosteric modulator of KRas4B signaling. Biophys J.

[291] Giancotti FG, Ruoslahti E (1999). Integrin signaling. Science.

[292] Humphries JD, Byron A, Bass MD, Craig SE, Pinney JW, Knight D, Humphries MJ (2009). Proteomic analysis of integrin-associated complexes identifies RCC2 as a dual regulator of Rac1 and Arf6. Sci Signal.

[293] Legate KR, Fässler R (2009). Mechanisms that regulate adaptor binding to beta-integrin cytoplasmic tails. J Cell Sci.

[294] Shattil SJ, Kim C, Ginsberg MH (2010). The final steps of integrin activation: the end game. Nat Rev Mol Cell Biol.

[295] Calderwood DA (2004). Talin controls integrin activation. Biochem Soc Trans.

[296] Campbell ID, Ginsberg MH (2004). The talin-tail interaction places integrin activation on FERM ground. Trends Biochem Sci.

[297] Wolfenson H, Iskratsch T, Sheetz MP (2014). Early events in cell spreading as a model for quantitative analysis of biomechanical events. Biophys J.

[298] Ye F, Snider AK, Ginsberg MH (2014). Talin and kindlin: the one-two punch in integrin activation. Front Med.

[299] Anthis NJ, Wegener KL, Critchley DR, Campbell ID (2010). Structural diversity in integrin/talin interactions. Structure.

[300] Seibert C, Veldkamp CT, Peterson FC, Chait BT, Volkman BR, Sakmar TP (2008). Sequential tyrosine sulfation of CXCR4 by tyrosylprotein sulfotransferases. Biochemistry.

[301] Chabot DJ, Chen DS, Dimitrov DS, Broder CC (2000). N-linked glycosylation of CXCR4 masks coreceptor function for CCR5-dependent human immunodeficiency virus type 1 isolates. J Virol.

[302] Ahr B, Denizot M, Robert-Hermann V, Brelot A, Biard-Piechaczyk M (2005). Identification of the cytoplasmic domains of CXCr4 involved in Jak2 and STAT3 phosphorylation. J Biol Chem.

[303] Busillo JM, Armando S, Sengupta R, Meucci O, Bouvier M, Benovic JL (2010). Site-specific phosphorylation of CXCR4 is dynamically regulated by multiple kinases and results in differential modulation of CSCR4 signaling. J Biol Chem.

[304] Marchese A, Raiborg C, Santini F, Keen JH, Stenmark H, Benovic JL (2003). The E3 ubiquitin ligase AIP4 mediates ubiquitination and sorting of the G protein-coupled receptor CXCR4. Dev Cell.

[305] Ding CB, Yu WN, Feng JH, Luo JM (2015). Structure and function of Gab2 and its role in cancer. Mol Med Rep.

[306] Visconti L, Malagrino F, Pagano L, Toto A (2020). Understanding the mechanism of recognition of Gab2 by the N-SH2 domain of SHP2. Life.

[307] McDonald CB, Seldeen KL, Deegan BJ, Bhat V, Farooq A (2010). Assembly of the Sos1-Grb2-Gab1 ternary signaling complex is under allosteric control. Arch Biochem Biophys.

[308] Simister PC, Feller SM (2012). Order and disorder in large multi-site docking proteins of the Gab family—implications for signaling complex formation and inhibitor design strategies. Mol BioSyst.

[309] Cheng J, Zhong Y, Chen S, Sun Y, Huang L, Kang Y (2017). Gab2 mediates hepatocellular carcinogenesis by integrating multiple signaling pathways. FASEB J.

[310] Gerlach GJ, Carrock R, Stix R, Stollar EJ, Ball KA (2020). A disordered encounter complex is central to the yeast Abp1p SH3 domain binding pathway. PLoS Comput Biol.

[311] Hunzicker-DunnME Lopez-Biladeau B, Law NC, Fiedler SE, Carr DW, Maizels ET (2012). PKA and GAB2 play central roles in the FSH signaling pathways to PI3K and AKT in ovarian granulosa cells. Proc Natl Acad Sci USA.

[312] Wöhrle FU, Daly RJ, Brummer T (2009). Function, regulation and pathological roles of the Gab/DOS docking proteins. Cell Commun Signaling.

[313] Toto A, Bonetti D, De Simone A, Gianni S (2017). Understanding the mechanism of binding between Gab2 and the C-terminal SH3 domain from Grb2. Oncotarget.

[314] Wöhrle FU, Daly RJ, Brummer T (2009). How to Grb2 a Gab. Structure.

[315] Harkiolaki M, Tsirka T, Lewitzky M, Simister PC, Joshi D, Bird LE (2009). Distinct binding modes of two epitopes in Gab2 that interact with the SH3C domain of Grb2. Structure.

[316] Moran MF, Koch CA, Anderson D, Ellis C, England L, Martin GS (1990). Src homology region 2 domains direct protein-protein interactions in signal transduction. Proc Natl Acad Sci USA.

[317] Bernstein A, Rossant J (2013). Anthony James Pawson (1952–2013). Nature.

[318] Kathiriya JJ, Pathak RR, Clayman E, Xue B, Uversky VN (2014). Davé. Presence and utility of intrinsically disordered regions in kinases. Mol BioSyst.

[319] Nussinov R, Tsai C-J, Jang H (2020). Ras assemblies and signaling at the membrane. Curr Opin Struct Biol.

[320] Nussinov R, Jang H, Tsai C-J, Liao T-J, Li S, Fushman D (2017). Intrinsic protein disorder in oncogenic KRAS signaling. Cell Mol Life Sci.

[321] Abdelkarim H, Banerjee A, Grudzien P, Leschinsky N, Abushaer M, Gaponenko V (2019). The hypervariable region of K-Ras4B governs molecular recognition and function. Int J Mol Sci.

[322] Hancock JF, Ras proteins,  (2003). Different signals from different locations. Mol Cell Biol.

[323] Minezaki Y, Homma K, Kinjo AR, Nishikawa R (2006). Human transcription factors contain a high fraction of intrinsically disordered regions essential for transcriptional regulation. J Mol Biol.

[324] Tsanev R, Vanatalu K, Jarvet J, Tanner R, Laur K, Tiigimägi P, Kragelund BB, Østerlund T, Kogerman P (2013). The transcriptional repressor domain of Gli3 is intrinsically disordered. PLoS ONE.

[325] Gomes T, Martin-Malpartida P, Ruiz L, Aragon E, Cordeiro TN, Macias MJ. Conformational landscape of full-length Smad proteins. BioRxiv. 10.1101/2021.04.13.439655. 10.1016/j.csbj.2021.09.009 PMC8479633 34630939

[326] Fang ZY, Takizawa N, Wilson KA, Smith TC, Delprato A, Davidson MW (2010). The membrane-associated protein, Supervillin, accelerate F-actin-dependent rapid integrin recycling an cell motility. Traffic.

[327] Snead D, Eliezer D (2019). Intrinsically disordered proteins in synaptic vesicle trafficking and release. J Biol Chem.

[328] Pietrosemoli N, Pancsa R, Tompa P (2013). Structural disorder provides increased adaptability for vesicle trafficking pathways. PLoS Comput Biol.

